# Adaptive Diversification of the *Mstnb* Locus Drives Genomic Architecture of Growth in Thai Clariid Catfish

**DOI:** 10.1111/eva.70324

**Published:** 2026-09-23

**Authors:** Ton Huu Duc Nguyen, Phonemany Thammachak, Trifan Budi, Piangjai Chalermwong, Chananya Patta, Wattanawan Jaito, Thitipong Panthum, Kednapat Sriphairoj, Sittichai Hatachote, Prapansak Srisapoome, Satid Chatchaiphan, Narongrit Muangmai, Orathai Sawatdichaikul, Agostinho Antunes, Kyudong Han, Darren K. Griffin, Jiraboon Prasanpan, Prateep Duengkae, Worapong Singchat, Kornsorn Srikulnath

**Affiliations:** ^1^ Animal Genomics and Bioresource Research Unit (AGB Research Unit), Faculty of Science Kasetsart University Bangkok Thailand; ^2^ Interdisciplinary Graduate Program in Bioscience, Faculty of Science Kasetsart University Bangkok Thailand; ^3^ School of Education, Faculty of Biology Education Can Tho University Can Tho Vietnam; ^4^ Department of Genetics, Faculty of Science Kasetsart University Bangkok Thailand; ^5^ School of Agricultural Technology King Mongkut's Institute of Technology Ladkrabang Bangkok Thailand; ^6^ Special Research Unit for Wildlife Genomics (SRUWG), Department of Forest Biology, Faculty of Forestry Kasetsart University Bangkok Thailand; ^7^ Department of Aquaculture, Faculty of Fisheries Kasetsart University Bangkok Thailand; ^8^ Department of Fishery Biology, Faculty of Fisheries Kasetsart University Bangkok Thailand; ^9^ Department of Nutrition and Health Institute of Food Research and Product Development, Kasetsart University Bangkok Thailand; ^10^ CIIMAR/CIMAR, Terminal de Cruzeiros Do Portode Leixões, Interdisciplinary Centre of Marine and Environmental Research University of Porto Porto Portugal; ^11^ Department of Biology, Faculty of Sciences University of Porto Porto Portugal; ^12^ Department of Microbiology Dankook University Cheonan Republic of Korea; ^13^ Bio‐Medical Engineering Core Facility Research Center Dankook University Cheonan Republic of Korea; ^14^ School of Biosciences University of Kent Canterbury UK; ^15^ Kalasin Fish Hatchery Farm (Betagro Public Company Limited) Kalasin Thailand; ^16^ Biodiversity Center Kasetsart University (BDCKU) Bangkok Thailand

**Keywords:** anthropogenic selection, evolutionary constraint, growth modulation, purifying selection, sustainable intensification

## Abstract

The production of clariid catfish in Thai aquaculture depends on culture of the native species ( *Clarias macrocephalus* and *C. batrachus* ) and introduced African catfish ( *C. gariepinus* ). Particularly, hybridization with *C. gariepinus* has improved productivity but raised significant conservation concerns. These lineages show distinct growth variations; however, the underlying genetic factors remain uncharacterized. We performed a comprehensive genomic assessment of the myostatin‐b (*Mstnb*) gene in 535 individuals comprising 19 Thai populations. The study design involved a novel integration of high‐throughput sequencing, structural modeling, and multi‐locus analysis. We identified eight novel alleles defined by 19 variable sites within exon 1. The observed diversity challenges the paradigm of extreme sequence conservation that typically occurs in vertebrate Myostatin. Although Bayesian phylogenetics showed intermingled allelic lineages, an integrated “growth‐gene pool” approach that combined *Mstnb* and *GH1* polymorphisms resolved a robust species‐level split (*K* = 2) and hierarchical sub‐structuring (*K* = 6). Selection analyses identified significant adaptive diversification (*ω* = 1.857). Codon‐level models highlighted Site 95 as a primary target of pervasive diversifying selection. Moreover, a nonsense mutation that resulted in a premature stop codon was identified as the mechanism underlying the functional modulation of growth traits. These findings provide a high‐resolution molecular toolkit for marker‐assisted selection and invasive species monitoring, which would enhance the sustainability of clariid aquaculture and protect native biodiversity against the risks of anthropogenic hybridization.

## 1 Introduction

Clariid catfishes, namely, the African catfish ( *Clarias gariepinus* ), bighead catfish ( *C. macrocephalus* ), and walking catfish ( *C. batrachus* ), are foundational aquaculture species in Southeast Asia and critical components of food security in Thailand because they provide essential high‐quality proteins and polyunsaturated fatty acids. Additionally, they support a robust industry with an annual Thai production of approximately 100,000 tons and a market value that exceeds US$150 million (Phetsang et al. 2021). Distinct growth rates among clariid species are well documented. Specifically, *C. gariepinus* exhibits a significantly faster growth rate than those of *C. macrocephalus* and *C. batrachus* (Patta et al. 2024; Srikulnath, Budi, et al. 2025). These advantageous phenotypic characteristics were combined by hybridizing female *C. macrocephalus* and male *C. gariepinus* to promote rapid growth with disease resistance. The resulting hybrids dominate > 90% of Thailand's catfish production (Patta et al. 2024; Srikulnath, Budi, et al. 2025). Although these breeding programs have historically enhanced productivity, they have simultaneously enabled a reliance on a narrow genetic base, which has led to declines in survival and fertility because of inbreeding depression. Stock genetic diversity has significantly decreased in terms of allelic variation in the growth hormone (*GH1*) locus, with specific sequence variants being linked to superior growth phenotypes in commercial strains (Thammachak et al. 2025). Moreover, the widespread introduction of *C. gariepinus* and subsequent hybridization events have led to urgent conservation concerns because interspecific crosses threaten the genomic integrity of native species through introgressive hybridization (Parvez et al. 2022). Recent breakthroughs in catfish genomics, such as the development of chromosome‐level genome assemblies for both *C. gariepinus* and *C. macrocephalus* , have provided the high‐resolution architectural framework necessary to investigate these complex issues (Nguinkal et al. 2024; Andres et al. 2025). However, despite the availability of these genomic resources, the specific allelic diversity and genetic mechanisms that regulate muscle development remain poorly understood. Therefore, characterizing the distribution of allelic variants at the genic level is imperative to identify molecular markers to mitigate the effects of genetic erosion and sustain industrial productivity (Lisachov et al. 2023; Patta et al. 2024; Srikulnath, Panthum, et al. 2025).

Myostatin (*Mstn*), otherwise known as the growth differentiation factor 8 (GDF‐8), is a highly conserved member of the transforming growth factor‐β (TGF‐β) superfamily. Mstn functions as a potent negative regulator of skeletal muscle development. Loss‐of‐function mutations associated with this regulator result in the “double‐muscled” phenotype in cattle and almost three‐fold increases in muscle mass in *mstn*‐null mice (McPherron and Lee 1997), which indicate the biological significance of this regulator. At the molecular level, mstn signals through activin Type II receptors (ActRIIB) to inhibit myoblast proliferation and differentiation, which maintains muscle homeostasis by regulating myogenic regulatory factors (Khalil et al. 2017). Unlike mammalian lineages, teleost fish have undergone lineage‐specific genome duplications, which have resulted in multiple *Mstn* paralogs (≤ 4 in salmonids) with partitioned expression profiles in different tissues such as the brain, intestine, gonads, and liver (Peñaloza et al. 2013; Khalil et al. 2017). Functional polymorphisms among these paralogues have been linked to economically important growth traits. For example, a single nucleotide polymorphism (SNP) in the 5′ flanking region of the *Mstn1b* gene of the Atlantic salmon is significantly associated with harvest weight, which accounts for the substantial phenotypic variation in body size (Peñaloza et al. 2013). Recent studies on other Siluriformes suggest that the *Mstnb* paralog may play a specialized role in regulating muscle fiber hyperplasia, which directly influences the final fillet yield and somatic growth rate. However, despite the recognized importance of the myostatin system, aquaculture genetics research has disproportionately focused on growth hormone (*GH1*) and insulin‐like growth factor (*IGF*) axes (Fuentes‐Lopez et al. 2020; Naya‐Català et al. 2021; Thammachak et al. 2025). Consequently, the *Mstnb* paralog in clariid catfish remains mostly uncharacterized and remains a significant knowledge gap in our understanding of catfish growth regulation. Therefore, characterizing the genetic variation in *Mstnb* is essential for identifying the molecular markers linked to superior performance and elucidating the evolutionary dynamics that govern this key growth regulator in different *Clarias* species (Segev‐Hadar et al. 2020; Shoyombo et al. 2022).

This study characterizes the genetic diversity, population architecture, and molecular selection signatures of the *Mstnb* gene across *C. gariepinus* , *C. macrocephalus* , and *C. batrachus* populations in Thailand. We hypothesized that intensive anthropogenic selection for growth in commercial catfish stocks has led to the emergence of lineage‐specific *Mstnb* variants, which are under significant selective pressure compared with that of wild populations. We have tested this hypothesis by addressing three primary research questions: (i) How is the allelic variation within *Mstnb* exon 1 partitioned across distinct clariid species and geographic locations? (ii) Are detectable signatures of selective sweeps or adaptive evolution available to distinguish the high‐growth commercial strains?, and (iii) Do the identified sequence variants influence the structural conservation of Myostatin‐b? We assessed the spatial distribution of growth‐related polymorphisms by sequencing the *Mstnb* exon 1 region to identify novel allelic lineages and quantify genetic variability. Population differentiation was evaluated by performing analysis of molecular variance (AMOVA) and Bayesian clustering, which provided a high‐resolution view of genomic subdivisions in the Thai catfish gene pool. Notably, we performed an integrated clustering analysis that combined the newly identified *Mstnb* variants with previously established growth hormone (*GH1*) polymorphism data (Thammachak et al. 2025) to establish a comprehensive “growth gene pool” framework for evaluating the cumulative genetic architecture of these commercial stocks. Moreover, we performed multi‐model selection tests such as Mixed Effects Model of Evolution (MEME), Fixed Effects Likelihood (FEL), and Fast Unconstrained Bayesian Approximation (FUBAR) to detect deviations from neutral evolution that may signify adaptive molecular shifts. We believe that the resulting data would provide robust molecular markers for selective breeding programs that aim to enhance somatic growth while simultaneously enabling the genetic management and conservation of native clariid populations. Thus, this study integrates fundamental molecular evolution with applied breeding strategies for promoting the long‐term productivity and resilience of a sustainable aquaculture industry in Southeast Asia.

## 2 Materials and Methods

### 2.1 Ethical Statement and Specimen Acquisition

In total, 535 *Clarias* specimens comprising five African catfish ( *C. gariepinus* ) populations, 13 bighead catfish ( *C. macrocephalus* ) populations, and one walking catfish ( *C. batrachus* ) population were collected from 19 geographically distinct locations across Thailand (Table S1). These specimens included both captive and wild individuals. They were sampled with the formal consent of the fish farm owners and relevant authorities. The caudal fin clips (approximately 0.3 × 0.3 cm) were collected non‐lethally, and all individuals were promptly released back to their natural habitats. The protocols used for animal handling in this study complied with the ARRIVE guidelines and were reviewed and approved by the Animal Experiment Committee at Kasetsart University (approval numbers: ACKU65‐SCI‐003, ACKU66‐SCI‐006, and ACCU66‐SCI‐014). The collected tissue samples were preserved in 1.5‐mL microcentrifuge tubes containing 95% ethanol and stored at 4°C until required for genomic processing. Genomic DNA was isolated using the standard salting‐out protocol described by Supikamolseni et al. (2015). DNA suitability in terms of quality and quantity for downstream molecular analysis was evaluated by performing 1% (w/v) agarose gel electrophoresis and spectrophotometry using a NanoDrop 2000 Spectrophotometer (Thermo Fisher Scientific, Wilmington, DE, USA), respectively.

### 2.2 Targeted Amplification and High‐Throughput Library Preparation

The *Mstnb* gene was initially identified by screening in‐house whole‐genome sequencing data against homologous sequences retrieved from public databases that included representative catfish and other teleost species data (Table S2). Preliminary comparative analysis showed a genomic structure that contained three exons and two introns. Exon 1 exhibited higher nucleotide variation than the other exonic regions. Therefore, a 342‐bp fragment from exon 1 that contained a high density of informative polymorphic sites was selected as the target region for both allelic analysis and phylogenetic inference. Additionally, the first exon of *Mstnb*, which has been previously associated with teleost growth traits (Wringe et al. 2010), was targeted for amplification. Primers were designed based on the *C. gariepinus* chromosome 5 reference sequence (Accession: NC071104) as follows: Catfish_mstnb_E1_F1 (5′‐TTGCCGTAGATGATCTGCTC‐3′) and Catfish_mstnb_E1_R1 (5′‐GCTTCCATTCTTGGAGGTCA‐3′). To facilitate downstream multiplexing, the 5′ end of the forward primer was tagged with a unique 8‐bp sample‐specific barcode (Macrogen Inc., Seoul, Korea). For Polymerase Chain Reaction (PCR), a 15‐μL reaction mixture containing 50 ng of genomic DNA, 1× standard reaction buffer, 1.5 mM MgCl_2_, 0.2 mM dNTPs, 0.5 μM of each primer, and 0.5 U of *Taq* polymerase (Apsalagen Co. Ltd., Bangkok, Thailand) was used. The thermal cycling profile comprised an initial denaturation at 95°C for 10 min; 40 cycles of 95°C for 30 s, 60°C for 30 s, and 72°C for 30 s; and a final extension at 72°C for 5 min. The resulting amplicons were visualized using 1.5% agarose gel electrophoresis. To ensure high‐fidelity results and minimize the occurrence of false alleles, each sample was amplified in triplicates. In total, 92 samples were each amplified using a unique barcode primer set and were pooled into nine separate libraries. Subsequently, these pools were sequenced on the Illumina NovaSeq 6000 platform (Novogene Co. Ltd., Singapore) to generate high‐quality 2 × 250 bp paired‐end short‐read data for comprehensive variant analysis.

### 2.3 Sequence Data Curation and High‐Stringency Quality Filtering

The quality of the 342‐bp paired‐end reads was rigorously assessed using FastQC v0.11.9 (Andrews 2010), and only reads with Phred quality score (*q* value) > 20 were retained for downstream analysis. Then, individual *Mstnb* amplicon sequences were clustered and assigned to specific specimens using the AmpliSAS pipeline (Sebastian et al. 2016) to facilitate accurate allelic filtering in high‐throughput datasets. The impact of technical artifacts and sequencing stochasticity was mitigated by establishing a stringent minimum amplicon depth of 100 reads per locus, and the maximum number of alleles per individual was restricted to two in order to reflect the diploid nature of the *Clarias* genome (Catchen et al. 2013; Andres et al. 2025). To distinguish true biological alleles from sequencing noise, we applied the “degree of change” criterion based on sequencing depth (Lighten et al. 2014) while maintaining the remaining parameters at their default configurations. Genotyping was performed based on allelic frequency. Samples exhibiting dominant allele frequency > 80% were classified as homozygous, whereas individuals possessing two distinct alleles were categorized as heterozygous. The resulting sequences were subjected to BLASTn analysis against the National Center for Biotechnology Information (NCBI) database to verify their identity, which typically requires ≥ 90% sequence identity for species‐level confirmation (https://blast.ncbi.nlm.nih.gov/Blast.cgi). Finally, all sequences were aligned and mapped to the *C. gariepinus* *Mstnb* reference sequence (Accession: NC071100) using Geneious Prime v2025.0.2 (https://www.geneious.com). Furthermore, this platform was used to translate nucleotide sequences into their corresponding amino acids for detecting premature stop codons or significant structural variations that might influence growth phenotypes.

### 2.4 Genetic Variation Metrics and Statistical Inference of *Mstnb* Diversity

The molecular variation within the *Mstnb* locus was characterized by using DnaSP v6.12 (Rozas et al. 2017) to perform DNA polymorphism analyses of the aligned 342 bp exon 1 sequences. For each population, we estimated several diversity indices such as the number of polymorphic (segregating) sites (*S*), number of haplotypes (*h*), haplotype diversity (*Hd*), nucleotide diversity (*π*), and average number of nucleotide differences (*k*). Other genetic diversity parameters included allelic frequency, observed number of alleles (*N*_a_), effective number of alleles (*N*_ea_), and both observed (*H*_o_) and expected (*H*_e_) heterozygosity. These were calculated using GenAlEx v6.5 (Peakall and Smouse 2006). Additionally, this software was used to assess the fixation index (*F*), deviations from the Hardy–Weinberg equilibrium (HWE), and linkage disequilibrium, which are critical for identifying potential selective pressures or non‐random mating within the *Clarias* populations. To explore the genetic architecture and differentiation among populations, genetic differentiation (*F*_ST_) was estimated using FSTAT v2.9.3 (Goudet 1995) by following the specialized protocols reported by Budi et al. (2023). Inbreeding coefficients (*F*_IS_) and allelic richness (AR) were calculated in Arlequin v3.5.2.2 (Excoffier and Lischer 2010) to partition the total genetic variance among and within populations and elucidate the spatial distribution of *Mstnb* polymorphisms across Thailand. AMOVA was performed on 535 individuals across 19 populations of three *Clarias* species using the pegas package (https://cran.r‐project.org/web/packages/pegas/index.html) in R v4.5.1 (R Core Team 2026).

### 2.5 Phylogenetic Inference and Evolutionary Mapping of *Mstnb* Lineages

To investigate the evolutionary relationships of the *Mstnb* gene within the order Siluriformes, representative nucleotide sequences were retrieved from the NCBI database using BLASTn by applying stringency thresholds of > 70% sequence identity and > 85% query coverage. MAFFT v7.490 (Katoh and Standley 2013) was used to align these sequences with an alignment strategy that was optimized for protein‐coding datasets to maintain codon integrity. The most appropriate nucleotide substitution model was identified using ModelFinder (Kalyaanamoorthy et al. 2017), which selects the GTR + G model based on the Bayesian information criterion. Phylogenetic reconstruction was performed using a dual approach involving Bayesian inference (BI) to ensure topological consistency. BI analysis was performed using MrBayes v3.2.6 (Ronquist and Huelsenbeck 2003) in the Geneious Prime 2025.0.2 (https://www.geneious.com) environment. This analysis used five Markov Chain Monte Carlo (MCMC) chains spanning 2,000,000 generations sampled every 5000 generations. A 10% burn‐in (200,000 generations) was discarded to ensure a posterior convergence. The resulting phylogenies were rooted using an a priori‐selected non‐Siluriformes teleost outgroup and visualized using the Interactive Tree of Life v5 (Letunic and Bork 2021). Finally, pairwise genetic distances among the *Mstnb* sequences were quantified using the Kimura 2‐parameter (K2P) model in MEGA 11, which provides a standardized metric for assessing interspecific and intraspecific molecular divergence.

### 2.6 Codon‐Based Selection Signatures and Locus‐Specific Evolutionary Constraints

The nature of the selective forces acting on the *Mstnb* gene was primarily assessed by estimating the *d*_N_/*d*_S_ ratio (*ω*), which measures the balance between non‐synonymous (*d*_N_) and synonymous (*d*_S_) substitution rates. The average values for *d*_N_ and *d*_S_ were calculated using the Nei–Gojobori method (Nei and Gojobori 1986) with a Jukes–Cantor correction in MEGA v12 (Kumar et al. 2024). Within this framework, *ω* ≈1 suggests neutral evolution, whereas *ω* > 1 and *ω* < 1 indicate positive and purifying selections, respectively. To further resolve the mode of selection at the population level, neutrality tests such as Tajima's *D*, Fu and Li's F*, and Fu and Li's D* were performed in DnaSP v6.12 (Rozas et al. 2017). The results helped identify deviations from neutral expectations. Population‐level signatures of non‐neutral evolution were evaluated by jointly considering *π*, *H*_d_, and neutrality statistics. Significantly negative neutrality statistics were interpreted as evidence of departure from neutral expectations, while acknowledging that similar patterns may also arise from demographic processes. *H*_e_ and the *F*_IS_ were retained as descriptors of within‐population genetic variation and heterozygote deficit or excess, respectively, and were not used as independent tests of selection. Additionally, we used the Datamonkey web server (http://datamonkey.org/) to execute three complementary codon‐level models that provided high‐resolution mapping of selective signatures: (i) Mixed EffectsModel of Evolutio (MEME), which detects the sites undergoing episodic diversifying selection (Murrell et al. 2012); (ii) Fixed EffectsLikelihood (FEL), which tests for pervasive selection across all lineages (Kosakovsky Pond and Frost 2005); and (iii) Fast Unconstrained Bayesian App Roximation (FUBAR), which uses a Bayesian framework to identify codons that evolve under pervasive positive or purifying selection (Murrell et al. 2013). Significance thresholds were strictly maintained at *p* ≤ 0.05 for likelihood‐based methods and posterior probability ≥ 0.9 for Bayesian inference.

### 2.7 Comparative Analysis of *Mstnb* Primary Structures and Residue Conservation

To evaluate the structural conservation of the *Mstnb* gene, orthologous amino acid sequences from diverse Siluriform species were retrieved from the NCBI database using BLASTp. This dataset included sequences from *C. macrocephalus* (JX456396), *C. gariepinus* (KJ372760, XM053497005), *Ameiurus catus* (AY540994), *Cranoglanis bouderius* (MH598837), *Ictalurus furcatus* (XM053626212, AY540992), *I. punctatus* (AF396747, XM017469117), *Pangasianodon hypophthalmus* (XM026946963), *Silurus lanzhouensis* (KU302769), *Tachysurus fulvidraco* (XM027171347, DQ767967), and *T*. *vachellii* (XM060877104). The BLASTp search used stringent thresholds of > 60% sequence identity and > 85% query coverage to ensure the inclusion of high‐quality orthologs for comparative analysis (Meiklejohn et al. 2019). The Mstnb amino acid residues identified in this study and the retrieved reference sequences were aligned using ClustalW in Geneious Prime v2025.0.2. After alignment, the sequences were trimmed to a conserved core of 100 residues, which acted as the basis for phylogenetic inference. Subsequently, a Bayesian phylogenetic tree was constructed with MrBayes v3.2.6 using a Markov Chain Monte Carlo (MCMC) analysis consisting of two independent runs. Each run was executed for 2,000,000 generations for the four chains, and sampling was performed every 1000 generations. A 25% burn‐in was applied to ensure the stability of the posterior probability estimates. The Mstnb protein secondary structures were predicted using the PSIPRED Workbench (https://bioinf.cs. ucl.ac.uk/psipred), and these were used to assess structural conservation and potential impact of amino acid substitutions on protein folding.

### 2.8 Assessment of Gene Pool Structure Through Integrated *Mstnb* and *GH1* Polymorphisms

The genetic structure of the sampled populations was inferred through a Bayesian clustering approach implemented in STRUCTURE v2.3.4 (Pritchard et al. 2000), which followed the multi‐locus methodology optimized by Budi et al. (2024). To enhance the resolution of our genomic assessment, we integrated the current *Mstnb* sequence data with our previously published diversity profiles of the *GH1* gene in *Clarias* species (Thammachak et al. 2025). The most probable number of genetic clusters (*K*) was determined by evaluating the log‐likelihood of the data (ln Pr (X|*K*)) across a range of *K* values. This enabled the identification of the point at which the likelihood stabilized. Additionally, the Δ*K* method was applied via Structure Selector (Li et al. 2019) to provide a statistically rigorous estimation of the optimal *K*. To complement the Bayesian inference, a Discriminant Analysis of Principal Components (DAPC) was executed using R v4.3.2 (R Core Team 2026) and the ADEGENET 2.0 package (Jombart 2008). This enabled the identification of genetic subdivisions without assuming Hardy–Weinberg equilibrium. Furthermore, pairwise genetic distances (*p*‐distance) for the 342‐bp *Mstnb* exon 1 sequences were calculated using MEGA 12 (Kumar et al. 2024). The resulting matrix served as the input for a Principal Coordinate Analysis (PCoA) performed in R to provide an exploratory visualization of sequence divergence. Final outputs were rendered using ggplot2. The individuals were color‐coded by species to illustrate the degree of sequence divergence and its correlation with population clustering (R Core Team 2026).

## 3 Results

### 3.1 Molecular Diversity and Genetic Architecture of *Mstnb* Locus in *Clarias* Populations

Sequence analysis of the 342‐bp *Mstnb* exon 1 fragment in 535 individuals showed 19 variable sites. Compared with that of the *C. gariepinus* reference sequence (NC071104), these sites defined eight newly identified alleles (Table S3). Genetic variation at the *Mstnb* exon 1 locus exhibited pronounced differences among the 19 sampled populations, and these were characterized at the sequence level to assess population‐specific diversity (Table S4). The number of segregating sites (*S*) ranged from 1 to 14. The highest *S* values were observed in SNK3‐CM‐W, KSN1‐CG‐C, and NYK‐CG‐C. *h* varied from 1–6, and the greatest diversity was detected in KSN1‐CG‐C and NYK‐CG‐C, whereas UBR‐CB‐C remained monomorphic. *Hd* ranged from 0.400 to 0.810, and was maximum in UBR‐CG‐C, followed closely by KSN1‐CG‐C. The lowest values were recorded in UBR‐CM‐C and SBR‐CG‐C. Furthermore, *k* showed substantial inter‐population variation ranging from 0.067 in NPT2‐CM‐W to 5.429 in UBR‐CG‐C. This highlights the broad range of genetic divergence across the study area. At the allelic level, *N*_a_ and *π* across all populations were 3.211 ± 0.311 and 0.004, respectively, which indicates relatively low nucleotide diversity despite the moderate number of alleles (Table 1). Furthermore, the mean AR was estimated as 3.313 ± 0.161, whereas the *N*_ea_ was 1.920 ± 0.115. The *H*_o_ and *H*_e_ averaged 0.493 ± 0.043 and 0.443 ± 0.036, respectively, with a statistically significant difference (*p* < 0.05) across the total dataset. Among the African catfish lineage, the KSN1‐CG‐C population exhibited the highest *H*_e_ content, whereas the SBR‐CG‐C population showed the lowest variation. By contrast, the bighead catfish showed the highest *H*_e_ in SNK4‐CM‐C, whereas UBR‐CM‐C exhibited the lowest diversity (Table 1). Although all populations conformed to Hardy–Weinberg equilibrium, the *F* varied spatially, and positive *F*‐values were recorded in five populations including KSN1‐CG‐C and SNK4‐CM‐C, whereas negative values were observed in 13 populations including NYK‐CG‐C and UBR‐CB‐C. *F*_ST_ between populations ranged from −0.329 to 0.910 (Table S5) with a mean *F*_IS_ of 0.056 ± 0.262. AMOVA was applied to partition the genetic diversity. We found that 82.59% of the total molecular variation was attributable to differences among species, whereas variation among populations nested within species accounted for only 2.16%. Variation among individuals within populations and within individuals accounted for 5.10% and 10.15% of the total variance, respectively (Table S6). Notably, the overall mean *H*_o_ and *H*_e_ comparison was significant; however, population‐specific pairwise comparisons remained inconclusive because of the fixation of alleles *H*_o_ = 0 in several groups and inherent constraints of analyzing a single locus (Tables S7–S9).

**TABLE 1:** Nucleotide sequence diversity in catfish populations based on *Mstnb* sequences.

Species	Population	Code	*N* ^a^	*N*_a_ ^b^	*N*_e_ ^c^	AR^d^	*H*_o_ ^e^	*H*_e_ ^f^	*F* ^g^	*F*_IS_ ^h^	*Π* ^i^	HWE^j^
*C. gariepinus*	Nakhon Nayok	NYK‐CG‐C	31	6.000	2.288	6.000	0.667	0.563	−0.203	−0.063	0.011	0.922^ns^
	Kalasin 1	KSN1‐CG‐C	91	6.000	2.997	6.000	0.615	0.666	0.076	0.090	0.009	0.000***
	Kalasin 2	KSN2‐CG‐C	139	3.000	1.814	3.000	0.489	0.449	−0.090	−0.083	0.002	0.505^ns^
	Sing Buri 1	SBR‐CG‐C	8	3.000	1.662	3.000	0.500	0.398	−0.255	−0.167	0.002	0.828^ns^
	Ubon Ratchathani 1	UBR‐CG‐C	4	4.000	2.909	4.000	0.750	0.656	−0.143	−0.080	0.018	0.677^ns^
	CGA	—	—	4.400 ± 0.678	2.334 ± 0.273	4.400 ± 0.678	0.606 ± 0.050	0.547 ± 0.054	−0.123 ± 0.057	−0.060	0.008	0.584^ns^
*C. macrocephalus*	Sing Buri 2	SBR‐CM‐C	18	3.000	1.710	3.000	0.556	0.415	−0.338	−0.192	0.002	0.447^ns^
	Sakon Nakhon 1	SNK1‐CM‐W	50	4.000	2.086	4.000	0.520	0.521	0.001	−0.018	0.003	0.000***
	Sakon Nakhon 2	SNK2‐CM‐W	81	4.000	1.879	4.000	0.407	0.468	0.129	0.351	0.002	0.000***
	Sakon Nakhon 3	SNK3‐CM‐W	24	4.000	2.091	4.000	0.708	0.522	−0.358	0.138	0.004	0.000***
	Sakon Nakhon 4	SNK4‐CM‐C	27	4.000	2.371	4.000	0.519	0.578	0.103	0.343	0.004	0.000***
	Suphan Buri 1	SPB1‐CM‐W	5	2.000	1.724	2.000	0.200	0.420	0.524	0.524	0.002	0.241^ns^
	Suphan Buri 2	SPB2‐CM‐W	3	2.000	1.800	2.000	0.667	0.444	−0.500	−0.500	0.002	0.386^ns^
	Nakhon Pathom 1	NPT1‐CM‐W	2	2.000	1.600	2.000	0.500	0.375	−0.333	−0.333	—	0.637^ns^
	Nakhon Pathom 2	NPT2‐CM‐W	2	2.000	1.600	2.000	0.500	0.375	−0.333	−0.333	—	0.637^ns^
	Nakhon Si Thammarat 1	NST1‐CM‐W	3	2.000	1.385	2.000	0.333	0.278	−0.200	−0.200		0.729^ns^
	Nakhon Si Thammarat 2	NST2‐CM‐C	10	4.000	2.151	4.000	0.600	0.535	−0.121	−0.128	0.003	0.807^ns^
	Surat Thani	STN‐CM‐C	26	3.000	2.129	3.000	0.577	0.530	−0.088	−0.027	0.002	0.124^ns^
	Ubon Ratchathani 2	UBR‐CM‐C	4	2.000	1.280	2.000	0.250	0.219	−0.143	0.261	0.001	0.775^ns^
	CMA	—	—	2.923 ± 0.262	1.831 ± 0.090	2.923 ± 0.262	0.487 ± 0.042	0.437 ± 0.029	−0.128 ± 0.075	−0.008	0.001	0.368^ns^
*C. batrachus*	Ubon Ratchathani 3	UBR‐CB‐C	7	1.000	1.000	1.000	0.000	0.000	—	0.286	0.000	—
	Overall	—	535	3.211	1.920	3.313	0.493	0.443	−0.126	0.056	0.004	—
	SD	—	—	±0.311	±0.115	±0.161	±0.043	±0.036	±0.054	0.262	—	—

Abbreviation: ns, not significant.

^a^ Number of individuals (*N*).

^b^ Number of different alleles (*N*_a_).

^c^ Number of effective alleles (*N*_e_).

^d^ Expected heterozygosity (*H*_e_).

^e^ Observed heterozygosity (*H*_o_).

^f^ Allelic Richness per locus and population (*AR*).

^g^ Fixation Index (*F*).

^h^ Inbreeding coefficient related to subpopulations (*F*_IS_).

^i^ Nucleotide diversity (π).

^j^ Hardy–Weinberg Equilibrium (HWE).

*** *p* < 0.001.

### 3.2 Systematic Mapping and Genealogical Relationships of *Mstnb* Allelic Lineages

**FIGURE 1:**
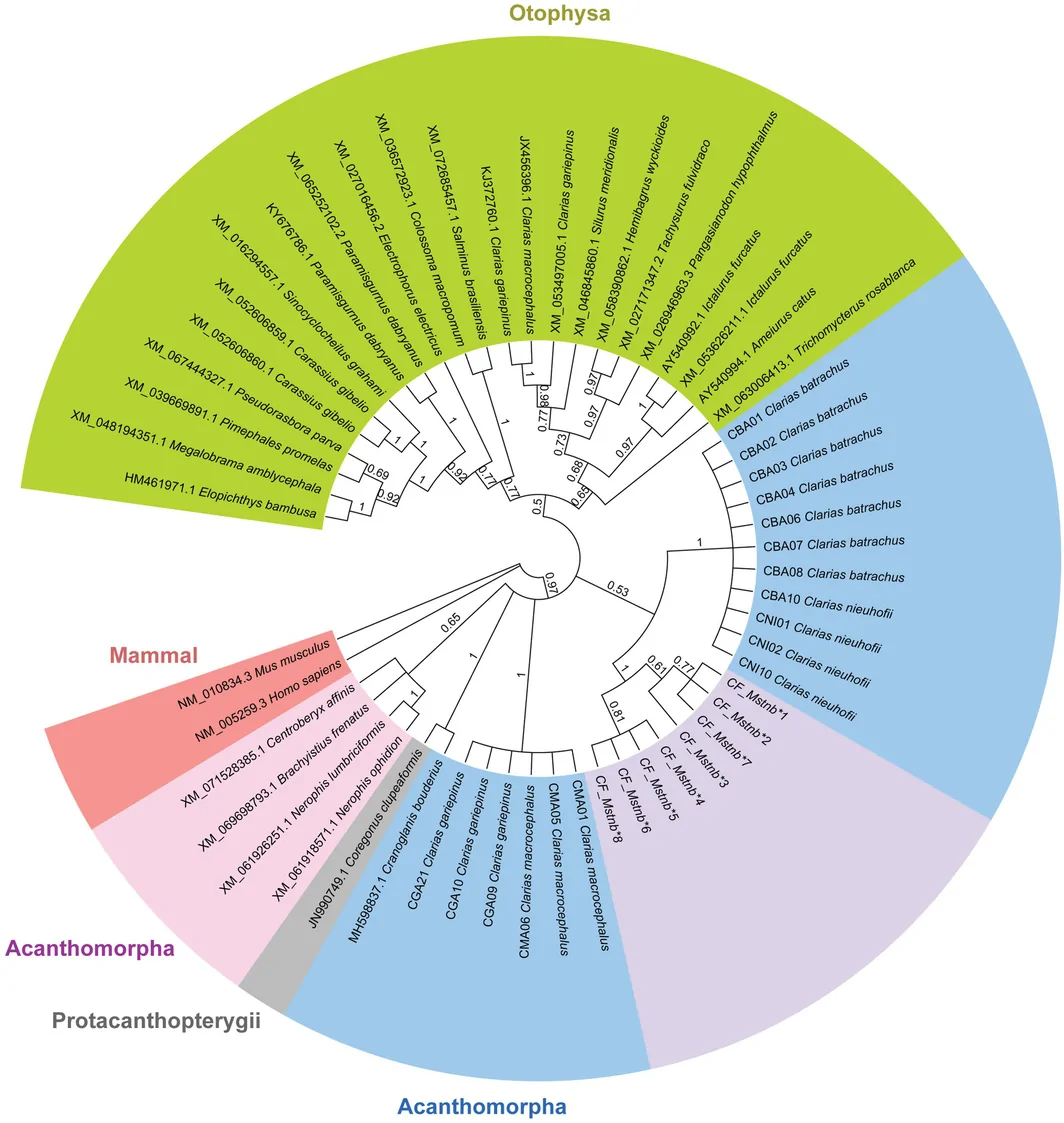
Phylogenetic tree of *Mstnb* genes reconstructed using 37 fish sequences, with two mammalian taxa included as outgroups. Colored sectors indicate major taxonomic groups: Red, Mammalia; pink, Acanthomorpha; lavender, Protacanthopterygii; and green, Otophysa. Within Otophysa, the target allele sequences (1–8) form a distinct clade sister to Siluriformes (catfishes), whereas other subgroups represent Cypriniformes (carps and minnows) and Characiformes (piranhas and tetras). Numbers at nodes indicate Bayesian posterior probabilities.

Phylogenetic inference based on Bayesian analysis indicated a lack of distinct species‐ or population‐based clustering among the *Mstnb* alleles in the African, bighead, and walking catfish populations. The eight sequence variants were further evaluated using a circular phylogenetic tree reconstructed via midpoint rooting to obtain a comprehensive view of the evolutionary trajectory of the gene (Figure 1). The phylogenetic tree was reconstructed using midpoint rooting to minimize the potential bias associated with overly distant outgroups. Representative teleost lineages outside the Otophysa, which included Acanthomorpha and Protacanthopterygii, were incorporated to provide a robust phylogenetic context. Additionally, mammalian sequences ( *Homo sapiens* and *Mus musculus* ) were included to solely function as external references and not the primary outgroups for rooting. Following the outgroup, the Acanthomorpha clade was recovered as a distinct lineage, whereas Protacanthopterygii formed a separate branch that was supported by a posterior probability of 0.88. The Otophysa clade constituted the largest group within the phylogeny with robust statistical support. Within this clade, two major lineages were recovered. The first comprised Cypriniformes, which included *Carassius gibelio* and *Pimephales promelas* . This group clustered into a well‐defined subclade that was supported by high posterior probabilities ranging from 0.91 to 1.00. Meanwhile, Siluriformes, which comprises representative clariid species, formed a separate and equally well‐supported lineage. This confirmed a distinct evolutionary divergence between these two major orders at the *Mstnb* locus. Thus, the eight *Mstnb* sequence variants grouped together as a monophyletic clade within the Siluriform lineage. This clade was resolved as a sister group to other *Clarias* sequences, with posterior probability values ranging from 0.65 to 0.81 (Figure 1).

### 3.3 Evidence of Non‐Neutral Evolution and Adaptive Divergence in *Mstnb* Locus

The selective pressures that act on *Mstnb* were evaluated based on the *d*_N_/*d*_S_ ratio (ω). We found signatures of positive selection in the African, bighead, and walking catfish populations (Table 2). The gene exhibited an average ω value of 1.857 (1.538–2.833), which indicates an overall trend toward adaptive diversification. The maximum *ω* value (2.833) was localized to three bighead catfish populations (SNK1‐CM‐W, SNK2‐CM‐W, and SNK4‐CM‐C). However, *ω* values remained undefined for 10 populations including KSN2‐CG‐C and UBR‐CB‐C. This may be attributed to the negligible frequency of *d*_S_, which prevented reliable ratio estimation (Table 3). Population‐level neutrality tests revealed heterogeneous departures from neutral expectations. SNK3‐CM‐W showed the strongest signal, with significantly negative Tajima's *D* (−2.020), Fu and Li's D* (−3.959), and Fu and Li's F* (−3.917). SNK1‐CM‐W also exhibited significantly negative Fu and Li's D* (−4.127) and F* (−3.823), whereas SNK4‐CM‐C showed significantly negative Fu and Li's D* (−2.791) and F* (−2.659). However, Tajima's *D* was not significant in SNK1‐CM‐W or SNK4‐CM‐C. These results indicate localized departures from neutral expectations, particularly in *C. macrocephalus* populations (Table 3). Nucleotide and haplotype diversity were not uniformly reduced across these populations. SNK1‐CM‐W, SNK3‐CM‐W, and SNK4‐CM‐C exhibited *Hd* values of 0.542, 0.545, and 0.629, respectively. Therefore, the observed neutrality‐test patterns were interpreted as localized signatures of non‐neutral evolution rather than definitive evidence of classical selective sweeps (Table S4).

**TABLE 2:** Rates of synonymous (*d*_S_) and non‐synonymous (*d*_N_) substitutions in nucleotide sequences of *Mstnb* gene in catfish populations.

Species	Population	Code	*N*	*d*_S_ (±SE)	*d*_N_ (±SE)	*ω* (*d*_N_ */d*_S_)	*Z*‐test	
							*p*	*Z*
*C. gariepinus*	Nakhon Nayok	NYK‐CG‐C	31	0.014 ± 0.010	0.019 ± 0.006	1.357	0.593	0.536
	Kalasin 1	KSN1‐CG‐C	91	0.014 ± 0.010	0.019 ± 0.007	1.357	0.565	0.578
	Kalasin 2	KSN2‐CG‐C	139	0.000 ± 0.000	0.003 ± 0.003	—	0.345	0.947
	Sing Buri 1	SBR‐CG‐C	8	0.000 ± 0.000	0.007 ± 0.005	—	0.188	1.323
	Ubon Ratchathani 1	UBR‐CG‐C	4	0.017 ± 0.013	0.022 ± 0.008	1.294	0.640	0.469
	CGA	—	273	0.014 ± 0.011	0.019 ± 0.007	1.357	0.586	0.546
*C. macrocephalus*	Sing Buri	SBR‐CM‐C	18	0.000 ± 0.000	0.007 ± 0.005	—	0.147	1.459
	Sakon Nakhon 1	SNK1‐CM‐W	50	0.006 ± 0.006	0.017 ± 0.006	2.833	0.187	1.328
	Sakon Nakhon 2	SNK2‐CM‐W	81	0.006 ± 0.006	0.017 ± 0.007	2.833	0.187	1.327
	Sakon Nakhon 3	SNK3‐CM‐W	24	0.015 ± 0.011	0.028 ± 0.008	1.866	0.263	1.124
	Sakon Nakhon 4	SNK4‐CM‐C	27	0.006 ± 0.006	0.017 ± 0.007	2.833	0.171	1.378
	Suphan Buri 1	SPB1‐CM‐W	5	0.000 ± 0.000	0.005 ± 0.005	—	0.330	0.977
	Suphan Buri 2	SPB2‐CM‐W	3	0.000 ± 0.000	0.005 ± 0.005	—	0.331	0.976
	Nakhon Pathom 1	NPT1‐CM‐W	2	0.000 ± 0.000	0.005 ± 0.005	—	0.318	1.004
	Nakhon Pathom 2	NPT2‐CM‐W	2	0.013 ± 0.009	0.020 ± 0.007	1.538	0.416	0.817
	Nakhon Si Thammarat 1	NST1‐CM‐W	3	0.000 ± 0.000	0.005 ± 0.005	—	0.338	0.962
	Nakhon Si Thammarat 2	NST2‐CM‐C	10	0.013 ± 0.009	0.020 ± 0.007	1.538	0.430	0.791
	Surat Thani	STN‐CM‐C	26	0.000 ± 0.000	0.007 ± 0.005	—	0.181	1.346
	Ubon Ratchathani 2	UBR‐CM‐C	4	0.000 ± 0.000	0.005 ± 0.005	—	0.315	1.008
	CMA	—	255	0.015 ± 0.010	0.027 ± 0.007	1.8	0.272	1.103
*C. batrachus*	Ubon Ratchathani 3	UBR‐CB‐C	7	—	—	—	—	—
	Overall mean value	—	535	0.014 ± 0.011	0.026 ± 0.008	1.857	0.271	1.106

**TABLE 3:** Neutrality tests of *Mstnb* gene sequences in catfish populations.

Species	Population	Code	Tajima's *D*	Fu and Li's *D*	Fu and Li's *F*
*C. gariepinus*	Nakhon Nayok	NYK‐CG‐C	0.786^ns^	0.932^ns^	1.043^ns^
	Kalasin 1	KSN1‐CG‐C	0.667^ns^	1.450^ns^	1.39^ns^
	Kalasin 2	KSN2‐CG‐C	1.049^ns^	0.635^ns^	0.905^ns^
	Sing Buri 1	SBR‐CG‐C	−0.532^ns^	−0.446^ns^	−0.53^ns^
	Ubon Ratchathani 1	UBR‐CG‐C	1.771^ns^	1.224^ns^	1.476^ns^
	CGA	—	0.748	0.759	0.856^ns^
*C. macrocephalus*	Sing Buri	SBR‐CM‐C	0.040^ns^	−0.714^ns^	−0.580^ns^
	Sakon Nakhon 1	SNK1‐CM‐W	−1.467^ns^	−4.127*	−3.823*
	Sakon Nakhon 2	SNK2‐CM‐W	−1.283^ns^	0.426^ns^	−0.193^ns^
	Sakon Nakhon 3	SNK3‐CM‐W	−2.020*	−3.959*	−3.917*
	Sakon Nakhon 4	SNK4‐CM‐C	−1.131^ns^	−2.791*	−2.659*
	Suphan Buri 1	SPB1‐CM‐W	0.851^ns^	1.052^ns^	1.029^ns^
	Suphan Buri 2	SPB2‐CM‐W	1.225^ns^	1.225^ns^	1.157^ns^
	Nakhon Pathom 1	NPT1‐CM‐W	—	—	—
	Nakhon Pathom 2	NPT2‐CM‐W	—	—	—
	Nakhon Si Thammarat 1	NST1‐CM‐W	−0.612^ns^	−0.612^ns^	−0.479^ns^
	Nakhon Si Thammarat 2	NST2‐CM‐C	0.558^ns^	0.935^ns^	0.953^ns^
	Surat Thani	STN‐CM‐C	0.748^ns^	0.771^ns^	0.886^ns^
	Ubon Ratchathani 2	UBR‐CM‐C	−0.817^ns^	−0.817^ns^	−0.772^ns^
	CMA	—	−0.355	−0.783	−0.763^ns^
*C. batrachus*	Ubon Ratchathani 3	UBR‐CB‐C	—	0.000^ns^	0.000^ns^
	Overall mean value	—	−0.005	−0.047	−0.055

Abbreviation: ns, not significant.

* *p* < 0.05.

Codon‐level analysis using the DataMonkey platform provided high‐resolution evidence of molecular selection. The MEME model identified Site 3 as undergoing episodic diversifying selection (*p* < 0.05), which may reflect transient adaptive shifts (Table S10). Although the FEL analysis detected no sites under pervasive selection (Table S11), the FUBAR model identified Site 95 as evolving under pervasive diversifying selection with a posterior probability of > 0.9 (Table S12). Further supported by high Bayes Factors, this site represents a significant functional constraint and is a primary candidate for future investigations on catfish growth modulation.

### 3.4 Functional Annotation and Secondary Structure Prediction of *Mstnb* Orthologs

Comparative analysis of the 100 amino acid residues of the *Mstnb* gene showed that sequence identities ranged from 70.97% to 100.00% across the eight newly identified alleles and various Siluriform species. Among the Thai catfish populations, we identified 18 missense mutations and one nonsense mutation that resulted in a premature stop codon in exon 1 (Table S13). The highest sequence identity (100%) was observed between the allele CF_Mstnb*01 and African catfish ( *Clarias gariepinus* , KJ372760) and between CF_Mstnb*02 and bighead catfish ( *C. macrocephalus* , JX456396). By contrast, the lowest similarity (70.97%) was recorded between CF_Mstnb*03 and Chinese catfish (*Tachysurus vachellii*, XM060877104). This result highlights the evolutionary distance between these siluriform lineages (Figure S1). Furthermore, these results indicate that *Mstnb* protein residues are highly conserved across the *Clarias* genus and related catfish species. This implies the maintenance of essential regulatory functions. Bayesian phylogenetic analysis based on these amino acid residues showed a polyphyletic distribution of *Mstnb* variants among the seven catfish species, which indicates a complex evolutionary history characterized by multiple ancestral lineages (Figure S2). Moreover, protein secondary structure prediction for *C. gariepinus* , *C. macrocephalus* , and *C. batrachus* showed that the three primary structural elements strands, helices, and coils constitute the fundamental folding architecture of Myostatin‐b (Figure S3).

### 3.5 Concordance of *Mstnb* and *GH1* Polymorphisms in Determining Catfish Population Structure

**FIGURE 2:**
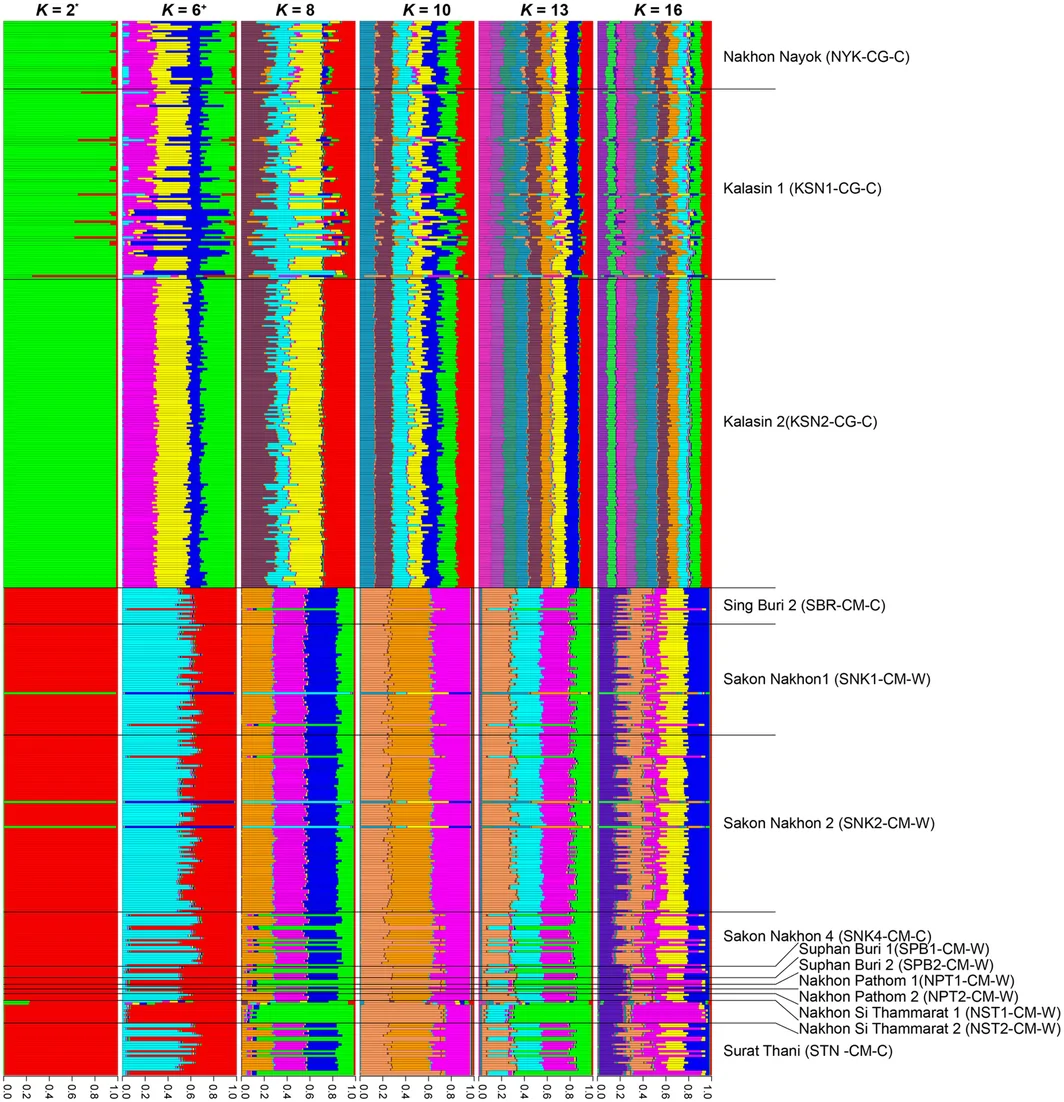
Bayesian clustering analysis of 5 African catfish, 13 bighead catfish, and 1 walking catfish populations in Thailand. The *x*‐axis shows the proportion of membership (posterior probability) in each genetic cluster represented by different color bars, and the *y*‐axis shows individuals within each population. The optimal number of clusters was observed at *K* = 2 based on Evanno's Δ*K* and ln Pr (*X*|*K*) strategies.

PCoA and DAPC multivariate analyses indicated the absence of distinct individual clustering within the broader dataset, which suggests a high degree of overlapping genetic variation at the microgeographic scale (Figures S4 and S5). By contrast, the Bayesian clustering analysis, which used a subset of individuals with both the *Mstnb* and *GH1* data available, consistently supported the presence of two primary genetic clusters (*K* = 2). This division was statistically supported by the maximum posterior probability Prob(*K* = 2) = 1, and it partitioned the samples into two distinct lineages: African catfish ( *C. gariepinus* ) and bighead catfish ( *C. macrocephalus* ) (Figure 2). Although the fundamental species‐level division remained stable, hierarchical sub‐structuring became apparent as *K* increased. This enabled a highly nuanced resolution of the population architecture within each species (Figure S6). Evanno's Δ*K* method identified *K* = 6 as the most pronounced level of secondary structure. This finding reflects fine‐scale differentiation within the two major species‐clades. This hierarchical pattern was consistently observed across the representative sampling locations, and it implied that although the primary species lineages were well preserved, localized genetic signatures were emerging at the sub‐population level.

## 4 Discussion

### 4.1 Evolutionary Constraints and Adaptive Diversification of *Mstnb* Locus

We identified eight novel *Mstnb* alleles in 535 individuals, which is indicative of a notable degree of molecular diversity. This challenges the paradigm of extreme sequence conservation that is typically observed in vertebrate Myostatin (McPherron and Lee 1997). Specifically, we identified 19 variable sites within the 342‐bp exon 1 fragment. This discovery contrasts with the limited polymorphism reported in Atlantic salmon (Peñaloza et al. 2013) and suggests that this locus in clariid catfish is particularly evolutionarily dynamic. Although the coding sequence remains under functional constraints, the high haplotype diversity and significant number of segregating sites indicate that exon 1 acts as a reservoir for adaptive variation. This inference was supported further by the *H*_e_ value, which reached its maximum in KSN1‐CG‐C and SNK4‐CM‐C, thereby indicating the presence of substantial genetic reservoirs for growth‐related traits. Moreover, the significant difference between *H*_o_ and *H*_e_ across the total dataset (*p* < 0.05) indicated that selective pressures or non‐random mating actively shifted the allelic equilibrium within these populations. Despite this molecular richness, Bayesian phylogenetic analysis showed a lack of species‐specific clustering, which indicates that allelic lineages are intermingled among the African, bighead, and walking catfish species. Furthermore, the nesting of target sequences within a broader clariid clade is indicative of this lack of resolution, and it likely reflects the slow evolution of the *Mstnb* coding sequence, which has maintained its regulatory integrity over a long evolutionary period (Sharma et al. 2015). However, the substantial spatial variation in diversity, such as the nucleotide differences reaching 5.429 in UBR‐CG‐C, highlights a complex landscape of localized genetic signatures that contrasts with the monomorphic nature of populations such as UBR‐CB‐C. AMOVA further demonstrated that genetic variation at the *Mstnb* locus was predominantly structured at the species level, with 82.59% of the total molecular variance occurring among species. By contrast, only 2.16% of the variance was attributable to populations within species, and this component was not statistically significant. This pattern indicates that differentiation at the *Mstnb* locus is primarily associated with species‐level divergence rather than pronounced geographic differentiation among populations within species. The strong species‐level partitioning detected by AMOVA, despite the absence of strictly species‐specific clustering in the allele phylogeny, suggests that species differ primarily in the distribution and frequencies of *Mstnb* variants rather than possessing completely exclusive allelic lineages. Although the overall nucleotide diversity remains relatively low, the presence of shared alleles and fluctuating *H*_e_ levels provide a unique opportunity for developing genetic markers to optimize growth performance in mixed‐species aquaculture (Ezilrani and Christopher 2015).

The results of the selection analyses of the three clariid species have revealed a complex evolutionary landscape where the average *d*_N_/*d*_S_ ratio (*ω* = 1.857) exceeded unity. This indicates that this locus is being actively shaped by episodes of positive selection. Additionally, this pattern differed significantly from our previous assessment of the *GH1* gene in *Clarias* species, which exhibited pervasive purifying selection (Thammachak et al. 2025). These contrasting signals suggest that *Mstnb* is a more flexible target for environmental or anthropogenic selection than *GH1*, which appears to be under stricter functional conservation. Significant negative neutrality statistics in SNK1‐CM‐W, SNK3‐CM‐W, and SNK4‐CM‐C indicated localized departures from neutral expectations, although demographic processes may produce similar patterns. Because nucleotide and haplotype diversity were not uniformly reduced, these signals were interpreted as evidence of non‐neutral evolution rather than definitive selective sweeps. This interpretation was further supported by FUBAR, which identified Site 95 as a candidate for pervasive diversifying selection. The maximum *ω* value (2.833) localized in bighead catfish populations suggests that localized adaptive events are occurring and that they likely reflect the aquaculture practices that favor growth‐enhancing variants (Sun et al. 2020). These findings concur with a previous conclusion that selective pressures modulate protein inhibitory function, which was inferred based on the significant association of specific myostatin polymorphisms with harvest weight in Atlantic salmon (Peñaloza et al. 2013). Furthermore, neutrality tests that identified significant selective signatures only in specific populations such as SNK3‐CM‐W supported this spatial heterogeneity in evolutionary forces. Positive selection has occasionally been detected particularly in farmed *C. gariepinus* , which aligns with aquaculture practices that favor growth‐enhancing variants (Sun et al. 2020). Consequently, identifying alleles under positive or balancing selection provides a roadmap for marker‐assisted selection, which would facilitate sustainable enhancement of productivity while preserving clariid catfish genetic resources (Khalil et al. 2017; Zhang et al. 2023).

### 4.2 Locus‐Specific Selection and Functional Implications for Clariid Growth Modulation

Codon‐level analysis provided high‐resolution evidence of molecular selection. Particularly, the MEME and FUBAR models identified specific residues that were targeted by diversifying forces. Site 3 had undergone episodic diversifying selection, which likely reflects transient adaptive shifts, whereas Site 95 showed evolution under pervasive diversifying selection (*PP* > 0.9). Furthermore, Site 95 was supported by high Bayes Factors, and it represents a significant functional constraint; hence, it is a primary candidate for future investigations into catfish growth modulation. The presence of these signatures in bighead catfish populations that exhibited maximum ω values indicates localized adaptive events driven by environmental pressures or intensive aquaculture selection. In addition to these specific sites, the biological role of the *Mstnb* propeptide region is central to interpreting the functional consequences of the 18 missense mutations that were identified in this study. This propeptide region acts as a self‐regulatory domain that binds to the mature myostatin dimer to block its activation and prevent premature signaling. Substitutions within this region such as those found in the eight novel alleles may weaken this inhibitory binding and increase the pool of bioavailable myostatin. These perturbations may intensify the negative regulation of muscle development, which provides a plausible mechanistic basis for the correlation between specific pro‐peptide alleles and growth phenotypes (McPherron and Lee 1997).

Despite these variations, comparative analysis of the 100‐residue core showed high sequence conservation (70.97%–100.00%) in *Clarias*, which suggests the maintenance of essential regulatory functions. Protein secondary structure predictions confirmed that the fundamental folding architecture comprising strands, helices, and coils remained intact in *C. gariepinus* , *C. macrocephalus* , and *C. batrachus* . However, the identification of a nonsense mutation that resulted in a premature stop codon indicates a major structural shift that likely triggers a loss‐of‐function phenotype, which could be a compelling target for breeding programs. Collectively, these results suggest that although the *Mstnb* scaffold is evolutionarily stable, specific residues and disruptive mutations undergo rapid diversification, which may directly influence the growth efficiency of clariid catfish.

### 4.3 Multi‐Locus Synergy: Integrating *Mstnb* and *GH1* to Resolve Population Architecture

The integration of *Mstnb* and *GH1* polymorphisms provided a high‐resolution perspective of the population structure and overcame the limitations of single‐locus assessments. Although *Mstnb* alone showed overlapping variations at the microgeographic scale, which reflected deep functional constraints and stabilizing selection that preserve its regulatory role (Gao et al. 2016), the combined dataset distinctly partitioned the samples into two primary clusters (*K* = 2). This robust split exactly corresponded to the *C. gariepinus* and *C. macrocephalus* lineages and is statistically supported by maximum posterior probabilities. These findings show that although *Mstnb* alleles intermingle phylogenetically, the incorporation of allelic frequency information from conserved loci provides a significant demographic signal that could resolve evolutionary independence. This cross‐gene analysis indicated distinct evolutionary forces, and *Mstnb* captured stabilizing selection, whereas *GH1* reflected anthropogenic pressures. Additionally, the *Mstnb* locus remained consistent across both wild and hatchery populations and showed low variation; in contrast, growth‐related endocrine loci such as *GH1* are typically more responsive to selection in aquaculture (Sherzada et al. 2023). Thus, artificial selection for faster growth in *C. gariepinus* broodstocks may introduce non‐neutral variation that produces moderate divergence among hatchery populations even in the absence of strong phylogenetic barriers (Abass et al. 2022). Moreover, populations under intensive farming tend to show shallow differentiation at *Mstnb* owing to their limited tolerance for disruptive mutations, whereas *GH1* signatures reflect the specific management background and breeding history of the stock. Furthermore, the identification of hierarchical sub‐structuring at *K* = 6 suggests that localized genetic signatures have begun to differentiate populations within these major species clades. This pattern was consistently observed across the 19 sampling locations and is likely driven by recent anthropogenically induced factors such as habitat fragmentation and intensive translocation of stocks. We integrated these datasets and obtained a realistic framework that aids in understanding population identity and consequences of breeding interventions. Additionally, it provides a multi‐locus perspective that is essential for detecting the differentiation that remains invisible under sequence‐based phylogenetics alone. Collectively, these insights helped determine the potential of using combined growth‐regulating markers to monitor genetic integrity, which could guide the sustainable management of Thai clariid resources.

### 4.4 Strategic Applications and Limitations: Connecting Genomic Data With Aquaculture and Conservation

Although the integrated approach used in this study provides high‐resolution insights into growth‐regulating loci, certain limitations must be acknowledged. The most significant challenge was the reliance on a single‐locus *Mstnb* fragment. Although exon 1 is a diversity hotspot, it may not capture the full extent of genome‐wide introgression or the complex polygenic nature of growth traits. Furthermore, the limited availability of matching *GH1* data for all individuals necessitated subsampling for Bayesian clustering, which may have omitted rare alleles present in the broader 535‐specimen dataset. Nevertheless, these constraints do not diminish the value of this study but rather highlight the specific utility of *Mstnb* as a high‐sensitivity diagnostic marker for identifying adaptive signatures that are otherwise invisible in broad‐scale genomic surveys. Future research should employ whole‐genome sequencing or high‐density SNP arrays to contextualize *Mstnb* within the broader genomic landscape. This will clarify synergistic growth pathways and ensure that low‐frequency alleles are fully captured across larger population scales.

Additionally, the identification of eight novel alleles and pervasive selection at Site 95 provides a robust molecular toolkit for marker‐assisted selection (MAS). This framework enables breeders to identify broodstocks that carry growth‐enhancing variants, which would streamline the development of high‐yield strains without the logistical burden of long‐term phenotypic trials (Khalil et al. 2017). Notably, the discovery of the nonsense mutation provides a definitive target for gene editing or selective breeding for muscle hypertrophy. In addition to production, the intermingled allelic lineages and hierarchical structure (*K* = 6) highlight the critical need for genetic monitoring during biological invasions. Often, *C. gariepinus* acts as an invasive species in local Thai watersheds, and its widespread translocation poses a severe threat to the genetic integrity of native *C. macrocephalus* and *C. batrachus* . The dual‐marker framework outlined in this paper may be used as a rapid diagnostic tool for detecting interspecific gene flow, which is essential for mitigating the ecological impact of alien species and preventing the “genetic swamping” of wild populations. Therefore, we propose that these *Mstnb* diversity profiles be used as a baseline for a national genetic registry to facilitate the traceability of farmed escapees and support the establishment of “genetic sanctuaries” for native clariids. We believe that these insights would bridge molecular evolution with practical policies through a dual strategy involving the maximizing of aquaculture productivity while safeguarding indigenous biodiversity against the risks of anthropogenic hybridization (Zhang et al. 2023).

## 5 Conclusion

This study has successfully characterized the *Mstnb* gene in various Thai clariid populations. This finding effectively fills the knowledge gap regarding the molecular regulation of catfish growth and supports the hypothesis that anthropogenic and environmental pressures have shaped *Mstnb* variation. Additionally, 19 variable sites have been identified, which indicates that this negative growth regulator undergoes more localized adaptive diversification than the extreme sequence conservation typically reported in vertebrates. Additionally, we have addressed our primary research questions. We have identified eight novel alleles in exon 1 and have observed that it is partitioned hierarchically into robust species‐level splits (*K* = 2) and localized sub‐structures (*K* = 6). Moreover, we have confirmed the presence of adaptive molecular shifts by detecting positive selection (*ω* = 1.857) and site‐specific signatures at Site 95. This finding helps distinguish high‐growth lineages and provides a suite of robust markers for selective breeding. The integration of *Mstnb* and *GH1* data was advantageous because it resulted in the establishment of a comprehensive “growth‐gene pool” framework that proved essential for resolving the population architecture, which remained obscured under single‐locus analysis. Moreover, we discovered a nonsense mutation and multiple missense substitutions. Although the core structural folding of the Myostatin‐b protein is preserved, this discovery highlights a distinct mechanism that underlies the functional modulation of growth traits. These results provide a powerful diagnostic toolkit for marker‐assisted selection and invasive species monitoring, which would enhance the productivity and resilience of the Southeast Asian aquaculture industry. Thus, this study combines molecular evolution with applied biotechnology to provide a definitive roadmap for the sustainable management of both commercial stocks and native clariid biodiversity.

## Funding

This research has received funding support from The Program Management Unit for Human Resources and Institutional Development and Innovation (PMU‐B) under the Program of National Postdoctoral and Postgraduate System (Contract No. B13F670053), which was awarded to W.S., T.P., N.M., P.D., and K.S. (Kornsorn Srikulnath); National Research Council of Thailand (NRCT): High‐Potential Research Team Grant Program (Contract number: N42A660605) awarded to W.S., T.P., K.S. (Kednapat Sriphairoj), S.H., N.M., P.D., and K.S. (Kornsorn Srikulnath); National Research Council of Thailand (NRCT) (Contract number: N42A650233) awarded to K.S. (Kornsorn Srikulnath); a grants from Betagro Group (No. 6501.0901.1/68) awarded to K.S. (Kornsorn Srikulnath); a Postdoctoral Fellowship from Kasetsart University awarded to T.P.; a grant from Kasetsart University Research and Development Institute (FF(KU) 61.69) awarded to W.S. and K.S. (Kornsorn Srikulnath); the Visiting Research Scholar (VRC) Grant, Faculty of Science, Kasetsart University (Contract Number VRC 4/2024), awarded to D.K.G. and K.S. (Kornsorn Srikulnath); The Office of the Ministry of Higher Education, Science, Research and Innovation; and the Thailand Science Research and Innovation (TSRI) through the Kasetsart University Reinventing University Program 2025 (No. RUP_Climate68_No. 9) awarded to W.S. and A.A.; a support from the International SciKU Branding (ISB), Faculty of Science, Kasetsart University awarded to T.P., W.S., and K.S. (Kornsorn Srikulnath); and the Thailand Scholarship (Scholarship number: MHESI 0202.3/10651) awarded to T.H.D.N. and K.S. (Kornsorn Srikulnath). No funding source was involved in the study design, collection, analysis, and interpretation of the data, writing of the report, or decision to submit the article for publication.

## Ethics Statement

All experimental procedures, including animal care, were reviewed and approved by the Animal Experiment Committee of Kasetsart University (approval no. ACKU65‐SCI‐003, ACKU66‐SCI‐006, and ACKU66‐SCI‐014) and were conducted in accordance with the Regulations on Animal Experiments of Kasetsart University ARRIVE https://arriveguidelines.org/, accessed on 8 September 2025.

## Conflicts of Interest

The authors declare no conflicts of interest.

## Supporting information


**Figure S1:** Alignment of partial amino acid sequences of the *Mstnb* gene (100 amino acids), covering the terminal region of exon 1, among eight allelic sequences found for 19 catfish populations (*C. gariepinus*, *C. macrocephalus* and *C. batrachus* ) in Thailand and reference sequences of 10 catfish species.
**Figure S2:** Bayesian phylogenetic tree based on *Mstnb* gene amino acid residues in catfish species.
**Figure S3:** eva70324‐sup‐0001‐AppendixS1.docx. *Mstnb* protein secondary structure production in North African catfish ( *C. gariepinus* ), bighead catfish ( *C. macrocephalus* ), and walking catfish ( *C. batrachus* ) in Thailand.
**Figure S4:** Principal component analysis (PCoA) of catfish populations in Thailand.
**Figure S5:** Discriminant analysis of principal components (DAPC) of five North African catfish and 13 bighead catfish and one walking catfish populations in Thailand. Each population is plotted by different colors.
**Figure S6:** Different population structure patterns of catfish populations in Thailand generated by the model‐based Bayesian clustering algorithms implemented in STRUCTURE based on (A) Plot of Evanno's Δ*K* and (B) Plot of Pr(X|*K*) strategy.
**Table S1:** Details of the catfish specimens used in this study.
**Table S2:** List of teleost and *Mstnb*‐related sequences retrieved from public databases used for comparative genomic analysis.
**Table S3:** Variable sites in the sequences of eight *Mstnb* alleles of North African catfish ( *C. gariepinus* ), bighead catfish ( *C. macrocephalus* ) and walking catfish ( *C. batrachus* ) populations in Thailand.
**Table S4:** Genetic diversity indices of catfish populations in Thailand based on the *Mstnb* sequences.
**Table S5:** Genetic differentiation between catfish populations in Thailand based on the *Mstnb* sequences
**Table S6:** The results of Analysis of Molecular Variance (AMOVA) for 19 catfish populations in Thailand.
**Table S7:** Comparison of observed (*H*_o_) and expected heterozygosity (*H*_e_) of the *Mstnb* gene in catfish populations.
**Table S8:** Comparison of expected heterozygosity (*H*_e_) of the *Mstnb* gene between catfish population.
**Table S9:** Comparison of observed heterozygosity (*H*_o_) for the *Mstnb* gene between catfish population.
**Table S10:** Detailed site‐by‐site results from the MEME analysis.
**Table S11:** Detailed site‐by‐site results from the FEL analysis.
**Table S12:** Codon sites under selection identified by FUBAR analysis.
**Table S13:** Single nucleotide substitutions and their functional effects in the partial fragment of the exon 1 of *Mstnb* gene of catfish populations in Thailand compare with the reference sequence (Accession number X NC_071104).

## Data Availability

All sequences were deposited in the National Center for Biotechnology Information (NCBI) (https://www.ncbi.nlm.nih.gov/; accession number: PX961328–PX961335 (accepted on 5 February 2026)).
