# Supplementary material for: Adaptive Diversification of the Mstnb Locus Drives Genomic Architecture of Growth in Thai Clariid Catfish
Source: Evol Appl. 2026 Sep 23;19(9):e70324. doi: 10.1111/eva.70324 (PMC13601680; doi:10.1111/eva.70324)
Supplement: Supplementary file 1 [file EVA-19-e70324-s001.docx]

Supplementary material

**Figure S1** Alignment of partial amino acid sequences of the *Mstnb* gene (100 amino acids), covering the terminal region of exon 1, among eight allelic sequences found for 19 catfish populations (*C gariepinus*, *C. macrocephalus* and *C*. *batrachus*) in Thailand and reference sequences of ten catfish species.

**Figure S2** Bayesian phylogenetic tree based on *Mstnb* gene amino acid residues in catfish species.

**Figure S3** Mstnb protein secondary structure production in North African catfish (*C. gariepinus*), bighead catfish (*C. macrocephalus*) and walking catfish (*C*. *batrachus)* in Thailand.

**Figure S4** Principal component analysis (PCoA) of catfish populations in Thailand

**Figure** **S5** Discriminant analysis of principal components (DAPC) of five North African catfish and 13 bighead catfish and one walking catfish populations in Thailand. Each population is plotted by different colors.

**Figure** **S6** Different population structure patterns of catfish populations in Thailand generated by the model-based Bayesian clustering algorithms implemented in STRUCTURE based on (A) Plot of Evanno's Δ*K* and (B) Plot of Pr(X|*K*) strategy

**Supplementary Table**

**Table S1** Details of the catfish specimens used in this study

| Species | Population | Code | *N*^1^ | Locations |  |  | GPS coordinate |
| --- | --- | --- | --- | --- | --- | --- | --- |
| *C. gariepinus* | Nakhon Nayok | NYK-CG-C | 31 | Jeed Jad Farm | Mueang Nakhon Nayok | Nakhon Nayok | 14˚05’58"N, 101˚09’39"E |
|  | Kalasin 1 | KSN1-CG-C | 91 | Kalasin Fish Farm (Betagro), Phu Sing | Mueang Kalasin | Kalasin | 16˚39’29"N, 103˚29’10"E |
|  | Kalasin 2 | KSN2-CG-C | 139 | Kalasin Fish Farm (Betagro), Phu Sing | Mueang Kalasin | Kalasin | 16˚39’29"N, 103˚29’10"E |
|  | Sing Buri 1 | SBR-CG-C | 8 | Phakin fish species | In Buri | Sing Buri | 14˚58’13"N, 100˚18’41"E |
|  | Ubon Ratchathani 1 | UBR-CG-C | 4 | Ubon Ratchathani University | Warin Chamrap | Ubon Ratchathani | 15˚7’5"N, 104˚54’5"E |
| *C. macrocephalus* | Sing Buri | SBR-CM-C | 18 | Phakin fish species | In Buri | Sing Buri | 14˚58’13"N, 100˚18’41"E |
|  | Sakon Nakhon 1 | SNK1-CM-W | 50 | Nong Han | Mueang Sakon Nakhon | Sakon Nakhon | 17°21’14"N, 104°19’16"E |
|  | Sakon Nakhon 2 | SNK2-CM-W | 81 | Susarn Donkajo | Mueang Sakon Nakhon | Sakon Nakhon | 17°14’05"N, 104°12’28"E |
|  | Sakon Nakhon 3 | SNK3-CM-W | 24 | Barnthavat | Mueang Sakon Nakhon | Sakon Nakhon | 17°14’46"N, 104°23’82"E |
|  | Sakon Nakhon 4 | SNK4-CM-C | 27 | Fisheries Research and Development center Sakon Nakhon | Mueang Sakon Nakhon | Sakon Nakhon | 17°17’06"N, 104°15’24"E |
|  | Suphan Buri 1 | SPB1-CM-W | 5 | Ban Chang Subdistrict | Song Phi Nong | Suphan Buri | 14°14’57.1"N, 100°11’13.0"E |
|  | Suphan Buri 2 | SPB2-CM-W | 3 | Khlong Bang Khan Chang, Bang Ta then subdistrict | Song Phi Nong | Suphan Buri | 14°14’40.5"N, 100°07’60.0"E |
|  | Nakhon Pathom 1 | NPT1-CM-W | 2 | Khlong Mon Thong | Kamphaeng Saen | Nakhon Pathom | 13°59’17.7"N, 99°56’37.3"E |
|  | Nakhon Pathom 2 | NPT2-CM-W | 2 | Tha Chin Rever Basin | Nakhon Chai Si | Nakhon Pathom | 13°88’01"N, 100°24’17"E |
|  | Nakhon Si Thammarat 1 | NST1-CM-W | 3 | River | Cha-uat | Nakhon Si Thammarat | 7°58’12"N, 99°58’12"E |
|  | Nakhon Si Thammarat 2 | NST2-CM-C | 10 | Nakhon Si Thammarat Pond | Phrom Khiri | Nakhon Si Thammarat | 8°61’27"N, 100°17’03"E |
|  | Surat Thani | STN-CM-C | 26 | Fisheries Center Surat Thani | Kanchanadit | Surat Thani | 9°15’92"N, 99°42’84"E |
|  | Ubon Ratchathani 2 | UBR-CM-C | 4 | Ubon Ratchathani University | Warin Chamrap | Ubon Ratchathani | 15˚7’5"N, 104˚54’5"E |
| *C*. *batrachus* | Ubon Ratchathani 3 | UBR-CB-C | 7 | Ubon Ratchathani University | Warin Chamrap | Ubon Ratchathani | 15˚7’5"N, 104˚54’5"E |

^1^ Number of samples (*N*)

**Table S2** List of teleost and *Mstnb*-related sequences retrieved from public databases used for comparative genomic analysis

| **No.** | **Species** | **Accession number** | **Clade** | **Country** |
| --- | --- | --- | --- | --- |
| 1 | *Brachyistius frenatus* | XM_069698793.1 | Acanthomorpha | Not specified (RefSeq) |
| 2 | *Centroberyx affinis* | XM_071528385.1 | Acanthomorpha | Not specified (RefSeq) |
| 3 | *Nerophis lumbriciformis* | XM_061926251.1 | Acanthomorpha | Not specified (RefSeq) |
| 4 | *Nerophis ophidion* | XM_061918571.1 | Acanthomorpha | Not specified (RefSeq) |
| 5 | *Ameiurus catus* | AY540994.1 | Otophysa | United States |
| 6 | *Carassius gibelio* | XM_052606860.1 | Otophysa | Not specified (RefSeq) |
| 7 | *Clarias gariepinus* | XM_053497005.1 | Otophysa | Not specified (RefSeq) |
| 8 | *Clarias gariepinus* | KJ372760.1 | Otophysa | China |
| 9 | *Clarias macrocephalus* | JX456396.1 | Otophysa | Thailand |
| 10 | *Colossoma macropomum* | XM_036572923.1 | Otophysa | Not specified (RefSeq) |
| 11 | *Cranoglanis bouderius* | MH598837.1 | Otophysa | China |
| 12 | *Electrophorus electricus* | XM_027016456.2 | Otophysa | Not specified (RefSeq) |
| 13 | *Elopichthys Bambusa* | HM461971.1 | Otophysa | China |
| 14 | *Hemibagrus wyckioides* | XM_058390862.1 | Otophysa | Not specified (RefSeq) |
| 15 | *Ictalurus furcatus* | XM_053626211.1 | Otophysa | Not specified (RefSeq) |
| 16 | *Ictalurus furcatus* | AY540992.1 | Otophysa | United States |
| 17 | *Megalobrama amblycephala* | XM_048194351.1 | Otophysa | Not specified (RefSeq) |
| 18 | *Pangasianodon hypophthalmus* | XM_026946963.3 | Otophysa | Not specified (RefSeq) |
| 19 | *Paramisgurnus dabryanus* | XM_065252102.2 | Otophysa | Not specified (RefSeq) |
| 20 | *Paramisgurnus dabryanus* | KY676786.1 | Otophysa | China |
| 21 | *Pimephales promelas* | XM_039669891.1 | Otophysa | Not specified (RefSeq) |
| 22 | *Pseudorasbora parva* | XM_067444327.1 | Otophysa | Not specified (RefSeq) |
| 23 | *Salminus brasiliensis* | XM_072685457.1 | Otophysa | Not specified (RefSeq) |
| 24 | *Silurus meridionalis* | XM_046845860.1 | Otophysa | Not specified (RefSeq) |
| 25 | *Sinocyclocheilus grahami* | XM_016294557.1 | Otophysa | Not specified (RefSeq) |
| 26 | *Tachysurus fulvidraco* | XM_027171347.2 | Otophysa | Not specified (RefSeq) |
| 27 | *Trichomycterus rosablanca* | XM_063006413.1 | Otophysa | Not specified (RefSeq) |
| 28 | *Coregonus clupeaformis* | JN990749.1 | Protacanthopterygii | Canada |

**Table S****3** Variable sites in the sequences of eight *Mstnb* alleles of North African catfish (*C. gariepinus*), bighead catfish (*C. macrocephalus*) and walking catfish (*C*. *batrachus)* populations in Thailand

| **Nucleotide position in the *Mstnb* gene of *Clarias gariepinus* (NC_071104)** | | **Variable sites of alleles** | | | | | | | |
| --- | --- | --- | --- | --- | --- | --- | --- | --- | --- |
|  |  | *CF_Mstnb*01* | *CF_Mstnb***02* | *CF_Mstnb***03* | *CF_Mstnb***04* | *CF_Mstnb***05* | *CF_Mstnb***06* | *CF_Mstnb***07* | *CF_Mstnb***08* |
| 36,326,310 | G | . | . | A | . | . | . | . | . |
| 36,326,319 | A | G | G | G | . | . | . | G | . |
| 36,326,334 | A | . | . | . | . | . | . | . | T |
| 36,326,361 | G | A | A | A | . | . | . | A | . |
| 36,326,367 | A | . | . | G | . | . | . | . | . |
| 36,326,379 | G | . | . | . | . | . | . | A | . |
| 36,326,391 | C | T | T | T | . | . | . | T | . |
| 36,326,396 | G | A | A | A | . | . | . | A | . |
| 36,326,406 | T | C | C | C | C | C | C | C | C |
| 36,326,426 | A | C | C | C | . | . | . | C | . |
| 36,326,469 | T | C | C | C | . | . | . | C | . |
| 36,326,484 | G | . | A | . | . | . | . | . | . |
| 36,326,496 | G | . | . | . | . | . | A | . | . |
| 36,326,511 | C | . | . | T | . | . | . | . | . |
| 36,326,517 | G | . | . | T | . | . | . | . | . |
| 36,326,559 | G | . | . | A | . | . | . | . | . |
| 36,326,568 | T | C | C | . | . | . | . | C | . |
| 36,326,579 | T | G | G | . | . | . | . | G | . |
| 36,326,589 | G | . | . | . | . | A | . | . | . |
| *N* | - | 250 | 163 | 12 | 251 | 100 | 6 | 37 | 53 |

*N*, Number of samples

**Table S4** Genetic diversity indices of catfish populations in Thailand based on the *Mstnb* sequences

| **Species** | **Population** | **Code** | ***S*^1^** | ***h*^2^** | ***Hd*^3^** | ***k*^4^** |
| --- | --- | --- | --- | --- | --- | --- |
| ***C. gariepinus*** | Nakhon Nayok | NYK-CG-C | 12 | 6 | 0.627 | 3.366 |
|  | Kalasin 1 | KSN1-CG-C | 12 | 6 | 0.709 | 2.712 |
|  | Kalasin 2 | KSN2-CG-C | 2 | 3 | 0.535 | 0.600 |
|  | Sing Buri 1 | SBR-CG-C | 2 | 3 | 0.473 | 0.505 |
|  | Ubon Ratchathani 1 | UBR-CG-C | 10 | 4 | 0.810 | 5.429 |
| ***C. macrocephalus*** | Sing Buri | SBR-CM-C | 2 | 3 | 0.500 | 0.524 |
|  | Sakon Nakhon 1 | SNK1-CM-W | 9 | 5 | 0.542 | 0.859 |
|  | Sakon Nakhon 2 | SNK2-CM-W | 8 | 4 | 0.500 | 0.692 |
|  | Sakon Nakhon 3 | SNK3-CM-W | 14 | 4 | 0.545 | 1.168 |
|  | Sakon Nakhon 4 | SNK4-CM-C | 8 | 4 | 0.629 | 1.124 |
|  | Suphan Buri 1 | SPB1-CM-W | 1 | 2 | 0.533 | 0.533 |
|  | Suphan Buri 2 | SPB2-CM-W | 1 | 2 | 0.600 | 0.600 |
|  | Nakhon Pathom 1 | NPT1-CM-W | 1 | 2 | 0.667 | 0.667 |
|  | Nakhon Pathom 2 | NPT2-CM-W | 1 | 2 | 0.667 | 0.067 |
|  | Nakhon Si Thammarat 1 | NST1-CM-W | 1 | 2 | 0.500 | 0.500 |
|  | Nakhon Si Thammarat 2 | NST2-CM-C | 2 | 3 | 0.560 | 0.758 |
|  | Surat Thani | STN-CM-C | 2 | 3 | 0.574 | 0.656 |
|  | Ubon Ratchathani 2 | UBR-CM-C | 1 | 2 | 0.400 | 0.400 |
| ***C*. *batrachus*** | Ubon Ratchathani 3 | UBR-CB-C | - | 1 | - | - |
|  | Overall mean value | - | 4.944 | 3.211 | 0.576 | 1.176 |

^1^ Polymorphic sites (*S*); ^2^ Number of haplotypes (*h*); ^3^ Haplotype diversity (*Hd*); ^4^ Average number of nucleotide differences (*k*)

**Table S5** Genetic differentiation between catfish populations in Thailand based on the *Mstnb* sequences

| **Population 1** | **Population 2** | ***F*_ST_** |
| --- | --- | --- |
| SNK3-CM-W | UBR-CM-C | 0.327 |
| SNK3-CM-W | UBR-CB-C | 0.624 |
| SNK3-CM-W | UBR-CG-C | 0.195 |
| SNK3-CM-W | SBR-CG-C | 0.504 |
| SNK3-CM-W | SBR-CM-C | 0.008 |
| SNK3-CM-W | KSN1-CG-C | 0.329 |
| SNK3-CM-W | NYK-CG-C | 0.398 |
| SNK3-CM-W | SNK4-CM-C | 0.173 |
| SNK3-CM-W | SNK2-CM-W | –0.009 |
| SNK3-CM-W | SNK1-CM-W | 0.000 |
| SNK3-CM-W | SPB1-CM-W | 0.135 |
| SNK3-CM-W | SPB2-CM-W | 0.134 |
| SNK3-CM-W | NPT1-CM-W | 0.088 |
| SNK3-CM-W | NPT2-CM-W | 0.088 |
| SNK3-CM-W | NST1-CM-W | 0.017 |
| SNK3-CM-W | NST2-CM-W | 0.383 |
| SNK3-CM-W | STN-CM-C | 0.032 |
| SNK3-CM-W | KSN2-CG-C | 0.517 |
| UBR-CM-C | UBR-CB-C | 0.910 |
| UBR-CM-C | UBR-CG-C | 0.086 |
| UBR-CM-C | SBR-CG-C | 0.645 |
| UBR-CM-C | SBR-CM-C | 0.473 |
| UBR-CM-C | KSN1-CG-C | 0.415 |
| UBR-CM-C | NYK-CG-C | 0.461 |
| UBR-CM-C | SNK4-CM-C | 0.023 |
| UBR-CM-C | SNK2-CM-W | 0.368 |
| UBR-CM-C | SNK1-CM-W | 0.250 |
| UBR-CM-C | SPB1-CM-W | 0.574 |
| UBR-CM-C | SPB2-CM-W | 0.604 |
| UBR-CM-C | NPT1-CM-W | 0.630 |
| UBR-CM-C | NPT2-CM-W | 0.630 |
| UBR-CM-C | NST1-CM-W | 0.618 |
| UBR-CM-C | NST2-CM-W | 0.500 |
| UBR-CM-C | STN-CM-C | 0.405 |
| UBR-CM-C | KSN2-CG-C | 0.590 |
| UBR-CB-C | UBR-CG-C | 0.712 |
| UBR-CB-C | SBR-CG-C | 0.778 |
| UBR-CB-C | SBR-CM-C | 0.718 |
| UBR-CB-C | KSN1-CG-C | 0.504 |
| UBR-CB-C | NYK-CG-C | 0.599 |
| UBR-CB-C | SNK4-CM-C | 0.578 |
| UBR-CB-C | SNK2-CM-W | 0.623 |
| UBR-CB-C | SNK1-CM-W | 0.621 |
| UBR-CB-C | SPB1-CM-W | 0.791 |
| UBR-CB-C | SPB2-CM-W | 0.844 |
| UBR-CB-C | NPT1-CM-W | 0.898 |
| UBR-CB-C | NPT2-CM-W | 0.898 |
| UBR-CB-C | NST1-CM-W | 0.903 |
| UBR-CB-C | NST2-CM-W | 0.682 |
| UBR-CB-C | STN-CM-C | 0.625 |
| UBR-CB-C | KSN2-CG-C | 0.639 |
| UBR-CG-C | SBR-CG-C | 0.295 |
| UBR-CG-C | SBR-CM-C | 0.318 |
| UBR-CG-C | KSN1-CG-C | 0.138 |
| UBR-CG-C | NYK-CG-C | 0.158 |
| UBR-CG-C | SNK4-CM-C | 0.021 |
| UBR-CG-C | SNK2-CM-W | 0.255 |
| UBR-CG-C | SNK1-CM-W | 0.165 |
| UBR-CG-C | SPB1-CM-W | 0.319 |
| UBR-CG-C | SPB2-CM-W | 0.302 |
| UBR-CG-C | NPT1-CM-W | 0.279 |
| UBR-CG-C | NPT2-CM-W | 0.279 |
| UBR-CG-C | NST1-CM-W | 0.311 |
| UBR-CG-C | NST2-CM-W | 0.308 |
| UBR-CG-C | STN-CM-C | 0.255 |
| UBR-CG-C | KSN2-CG-C | 0.325 |
| SBR-CG-C | SBR-CM-C | 0.579 |
| SBR-CG-C | KSN1-CG-C | 0.033 |
| SBR-CG-C | NYK-CG-C | 0.004 |
| SBR-CG-C | SNK4-CM-C | 0.468 |
| SBR-CG-C | SNK2-CM-W | 0.540 |
| SBR-CG-C | SNK1-CM-W | 0.519 |
| SBR-CG-C | SPB1-CM-W | 0.552 |
| SBR-CG-C | SPB2-CM-W | 0.550 |
| SBR-CG-C | NPT1-CM-W | 0.565 |
| SBR-CG-C | NPT2-CM-W | 0.565 |
| SBR-CG-C | NST1-CM-W | 0.611 |
| SBR-CG-C | NST2-CM-W | 0.495 |
| SBR-CG-C | STN-CM-C | 0.502 |
| SBR-CG-C | KSN2-CG-C | –0.011 |
| SBR-CM-C | KSN1-CG-C | 0.368 |
| SBR-CM-C | NYK-CG-C | 0.457 |
| SBR-CM-C | SNK4-CM-C | 0.268 |
| SBR-CM-C | SNK2-CM-W | –0.009 |
| SBR-CM-C | SNK1-CM-W | 0.041 |
| SBR-CM-C | SPB1-CM-W | 0.092 |
| SBR-CM-C | SPB2-CM-W | 0.105 |
| SBR-CM-C | NPT1-CM-W | 0.032 |
| SBR-CM-C | NPT2-CM-W | 0.032 |
| SBR-CM-C | NST1-CM-W | -0.048 |
| SBR-CM-C | NST2-CM-W | 0.433 |
| SBR-CM-C | STN-CM-C | 0.013 |
| SBR-CM-C | KSN2-CG-C | 0.557 |
| KSN1-CG-C | NYK-CG-C | 0.021 |
| KSN1-CG-C | SNK4-CM-C | 0.334 |
| KSN1-CG-C | SNK2-CM-W | 0.380 |
| KSN1-CG-C | SNK1-CM-W | 0.357 |
| KSN1-CG-C | SPB1-CM-W | 0.345 |
| KSN1-CG-C | SPB2-CM-W | 0.322 |
| KSN1-CG-C | NPT1-CM-W | 0.319 |
| KSN1-CG-C | NPT2-CM-W | 0.319 |
| KSN1-CG-C | NST1-CM-W | 0.363 |
| KSN1-CG-C | NST2-CM-W | 0.338 |
| KSN1-CG-C | STN-CM-C | 0.335 |
| KSN1-CG-C | KSN2-CG-C | 0.044 |
| NYK-CG-C | SNK4-CM-C | 0.365 |
| NYK-CG-C | SNK2-CM-W | 0.449 |
| NYK-CG-C | SNK1-CM-W | 0.417 |
| NYK-CG-C | SPB1-CM-W | 0.444 |
| NYK-CG-C | SPB2-CM-W | 0.427 |
| NYK-CG-C | NPT1-CM-W | 0.434 |
| NYK-CG-C | NPT2-CM-W | 0.434 |
| NYK-CG-C | NST1-CM-W | 0.464 |
| NYK-CG-C | NST2-CM-W | 0.414 |
| NYK-CG-C | STN-CM-C | 0.412 |
| NYK-CG-C | KSN2-CG-C | 0.026 |
| SNK4-CM-C | SNK2-CM-W | 0.231 |
| SNK4-CM-C | SNK1-CM-W | 0.133 |
| SNK4-CM-C | SPB1-CM-W | 0.318 |
| SNK4-CM-C | SPB2-CM-W | 0.289 |
| SNK4-CM-C | NPT1-CM-W | 0.291 |
| SNK4-CM-C | NPT2-CM-W | 0.291 |
| SNK4-CM-C | NST1-CM-W | 0.300 |
| SNK4-CM-C | NST2-CM-W | 0.277 |
| SNK4-CM-C | STN-CM-C | 0.223 |
| SNK4-CM-C | KSN2-CG-C | 0.505 |
| SNK2-CM-W | SNK1-CM-W | 0.017 |
| SNK2-CM-W | SPB1-CM-W | 0.118 |
| SNK2-CM-W | SPB2-CM-W | 0.105 |
| SNK2-CM-W | NPT1-CM-W | 0.027 |
| SNK2-CM-W | NPT2-CM-W | 0.027 |
| SNK2-CM-W | NST1-CM-W | –0.046 |
| SNK2-CM-W | NST2-CM-W | 0.421 |
| SNK2-CM-W | STN-CM-C | 0.029 |
| SNK2-CM-W | KSN2-CG-C | 0.541 |
| SNK1-CM-W | SPB1-CM-W | 0.187 |
| SNK1-CM-W | SPB2-CM-W | 0.175 |
| SNK1-CM-W | NPT1-CM-W | 0.130 |
| SNK1-CM-W | NPT2-CM-W | 0.130 |
| SNK1-CM-W | NST1-CM-W | 0.056 |
| SNK1-CM-W | NST2-CM-W | 0.393 |
| SNK1-CM-W | STN-CM-C | 0.066 |
| SNK1-CM-W | KSN2-CG-C | 0.529 |
| SPB1-CM-W | SPB2-CM-W | –0.200 |
| SPB1-CM-W | NPT1-CM-W | –0.329 |
| SPB1-CM-W | NPT2-CM-W | –0.329 |
| SPB1-CM-W | NST1-CM-W | –0.042 |
| SPB1-CM-W | NST2-CM-W | 0.248 |
| SPB1-CM-W | STN-CM-C | –0.007 |
| SPB1-CM-W | KSN2-CG-C | 0.547 |
| SPB2-CM-W | NPT1-CM-W | –0.176 |
| SPB2-CM-W | NPT2-CM-W | –0.176 |
| SPB2-CM-W | NST1-CM-W | 0.063 |
| SPB2-CM-W | NST2-CM-W | 0.203 |
| SPB2-CM-W | STN-CM-C | –0.026 |
| SPB2-CM-W | KSN2-CG-C | 0.535 |
| NPT1-CM-W | NPT2-CM-W | –0.333 |
| NPT1-CM-W | NST1-CM-W | –0.090 |
| NPT1-CM-W | NST2-CM-W | 0.258 |
| NPT1-CM-W | STN-CM-C | –0.080 |
| NPT1-CM-W | KSN2-CG-C | 0.543 |
| NPT2-CM-W | NST1-CM-W | –0.090 |
| NPT2-CM-W | NST2-CM-W | 0.258 |
| NPT2-CM-W | STN-CM-C | –0.080 |
| NPT2-CM-W | KSN2-CG-C | 0.543 |
| NST1-CM-W | NST2-CM-W | 0.437 |
| NST1-CM-W | STN-CM-C | –0.021 |
| NST1-CM-W | KSN2-CG-C | 0.573 |
| NST2-CM-W | STN-CM-C | 0.290 |
| NST2-CM-W | KSN2-CG-C | 0.512 |
| STN-CM-C | KSN2-CG-C | 0.521 |

**Table S6** The results of Analysis of Molecular Variance (AMOVA) for 19 catfish populations in Thailand.

| Source | df ^1^ | SS ^2^ | Var ^3^ | % of variance |
| --- | --- | --- | --- | --- |
| Among species | 2 | 18293.779 | 17.379 | 82.59% |
| Among populations within species | 16 | 439.642 | 0.454 | 2.16% |
| Among individuals within populations | 516 | 2208.959 | 1.073 | 5.10% |
| Within individuals | 235 | 1142.500 | 2.136 | 10.15% |
| Total | 1069 | 22084.879 | 21.041 | 100% |

^1^ Degree of freedom (df); ^2^ Sum of squares (SS); ^3^ Variance components (Var).

**Table S7** Comparison of observed (*H*_o_) and expected heterozygosity (*H*_e_) of the *Mstnb* gene in catfish populations

| **Populations** | **Difference of means** | **Standard Error** | ***t*-test** | ***p*-value** |
| --- | --- | --- | --- | --- |
| SNK3-CM-W | 0.186 | 0 | Inf | N/A |
| UBR-CM-C | 0.031 | 0 | Inf | N/A |
| UBR-CB-C | 0 | 0 | Inf | N/A |
| UBR-CG-C | 0.094 | 0 | Inf | N/A |
| SBR-CG-C | 0.102 | 0 | Inf | N/A |
| SBR-CM-C | 0.141 | 0 | Inf | N/A |
| KSN1-CG-C | –0.046 | 0 | Inf | N/A |
| NYK-CG-C | 0.114 | 0 | Inf | N/A |
| SNK4-CM-C | –0.059 | 0 | Inf | N/A |
| SNK2-CM-W | –0.061 | 0 | Inf | N/A |
| SNK1-CM-W | 0.019 | 0 | Inf | N/A |
| SPB1-CM-W | –0.22 | 0 | Inf | N/A |
| SPB2-CM-W | 0.223 | 0 | Inf | N/A |
| NPT1-CM-W | 0.125 | 0 | Inf | N/A |
| NPT2-CM-W | 0.125 | 0 | Inf | N/A |
| NST1-CM-W | 0.055 | 0 | Inf | N/A |
| NST2-CM-W | 0.065 | 0 | Inf | N/A |
| STN-CM-C | 0.047 | 0 | Inf | N/A |
| KSN2-CG-C | 0.04 | 0 | Inf | N/A |

Inf, infinitive; N/A, not applicable

**Table S8** Comparison of expected heterozygosity (*H*_e_) of the *Mstnb* gene between catfish population

| **Population 1** | **Population 2** | **Difference of means** | **Standard**  **Error** | ***t*-test** | ***p*-value** |
| --- | --- | --- | --- | --- | --- |
| SNK3-CM-W | UBR-CM-C | 0.303 | 0 | Inf | N/A |
| SNK3-CM-W | UBR-CB-C | 0.522 | 0 | Inf | N/A |
| SNK3-CM-W | UBR-CG-C | –0.134 | 0 | -Inf | N/A |
| SNK3-CM-W | SBR-CG-C | 0.124 | 0 | Inf | N/A |
| SNK3-CM-W | SBR-CM-C | 0.107 | 0 | Inf | N/A |
| SNK3-CM-W | KSN1-CG-C | –0.150 | 0 | -Inf | N/A |
| SNK3-CM-W | NYK-CG-C | –0.041 | 0 | -Inf | N/A |
| SNK3-CM-W | SNK4-CM-C | –0.056 | 0 | -Inf | N/A |
| SNK3-CM-W | SNK2-CM-W | 0.054 | 0 | Inf | N/A |
| SNK3-CM-W | SNK1-CM-W | 0.001 | 0 | Inf | N/A |
| SNK3-CM-W | SPB1-CM-W | 0.102 | 0 | Inf | N/A |
| SNK3-CM-W | SPB2-CM-W | 0.078 | 0 | Inf | N/A |
| SNK3-CM-W | NPT1-CM-W | 0.147 | 0 | Inf | N/A |
| SNK3-CM-W | NPT2-CM-W | 0.147 | 0 | Inf | N/A |
| SNK3-CM-W | NST1-CM-W | 0.244 | 0 | Inf | N/A |
| SNK3-CM-W | NST2-CM-W | –0.013 | 0 | -Inf | N/A |
| SNK3-CM-W | STN-CM-C | –0.008 | 0 | -Inf | N/A |
| SNK3-CM-W | KSN2-CG-C | 0.073 | 0 | Inf | N/A |
| UBR-CM-C | UBR-CB-C | 0.219 | 0 | Inf | N/A |
| UBR-CM-C | UBR-CG-C | –0.437 | 0 | -Inf | N/A |
| UBR-CM-C | SBR-CG-C | –0.179 | 0 | -Inf | N/A |
| UBR-CM-C | SBR-CM-C | –0.196 | 0 | -Inf | N/A |
| UBR-CM-C | KSN1-CG-C | –0.453 | 0 | -Inf | N/A |
| UBR-CM-C | NYK-CG-C | –0.344 | 0 | -Inf | N/A |
| UBR-CM-C | SNK4-CM-C | –0.359 | 0 | -Inf | N/A |
| UBR-CM-C | SNK2-CM-W | –0.249 | 0 | -Inf | N/A |
| UBR-CM-C | SNK1-CM-W | –0.302 | 0 | -Inf | N/A |
| UBR-CM-C | SPB1-CM-W | –0.201 | 0 | -Inf | N/A |
| UBR-CM-C | SPB2-CM-W | –0.225 | 0 | -Inf | N/A |
| UBR-CM-C | NPT1-CM-W | –0.156 | 0 | -Inf | N/A |
| UBR-CM-C | NPT2-CM-W | –0.156 | 0 | -Inf | N/A |
| UBR-CM-C | NST1-CM-W | –0.059 | 0 | -Inf | N/A |
| UBR-CM-C | NST2-CM-W | –0.316 | 0 | -Inf | N/A |
| UBR-CM-C | STN-CM-C | –0.311 | 0 | -Inf | N/A |
| UBR-CM-C | KSN2-CG-C | –0.230 | 0 | -Inf | N/A |
| UBR-CB-C | UBR-CG-C | –0.656 | 0 | -Inf | N/A |
| UBR-CB-C | SBR-CG-C | –0.398 | 0 | -Inf | N/A |
| UBR-CB-C | SBR-CM-C | –0.415 | 0 | -Inf | N/A |
| UBR-CB-C | KSN1-CG-C | –0.672 | 0 | -Inf | N/A |
| UBR-CB-C | NYK-CG-C | –0.563 | 0 | -Inf | N/A |
| UBR-CB-C | SNK4-CM-C | –0.578 | 0 | -Inf | N/A |
| UBR-CB-C | SNK2-CM-W | –0.468 | 0 | -Inf | N/A |
| UBR-CB-C | SNK1-CM-W | –0.521 | 0 | -Inf | N/A |
| UBR-CB-C | SPB1-CM-W | –0.420 | 0 | -Inf | N/A |
| UBR-CB-C | SPB2-CM-W | –0.444 | 0 | -Inf | N/A |
| UBR-CB-C | NPT1-CM-W | –0.375 | 0 | -Inf | N/A |
| UBR-CB-C | NPT2-CM-W | –0.375 | 0 | -Inf | N/A |
| UBR-CB-C | NST1-CM-W | –0.278 | 0 | -Inf | N/A |
| UBR-CB-C | NST2-CM-W | –0.535 | 0 | -Inf | N/A |
| UBR-CB-C | STN-CM-C | –0.530 | 0 | -Inf | N/A |
| UBR-CB-C | KSN2-CG-C | –0.449 | 0 | -Inf | N/A |
| UBR-CG-C | SBR-CG-C | 0.258 | 0 | Inf | N/A |
| UBR-CG-C | SBR-CM-C | 0.241 | 0 | Inf | N/A |
| UBR-CG-C | KSN1-CG-C | –0.016 | 0 | -Inf | N/A |
| UBR-CG-C | NYK-CG-C | 0.093 | 0 | Inf | N/A |
| UBR-CG-C | SNK4-CM-C | 0.078 | 0 | Inf | N/A |
| UBR-CG-C | SNK2-CM-W | 0.188 | 0 | Inf | N/A |
| UBR-CG-C | SNK1-CM-W | 0.135 | 0 | Inf | N/A |
| UBR-CG-C | SPB1-CM-W | 0.236 | 0 | Inf | N/A |
| UBR-CG-C | SPB2-CM-W | 0.212 | 0 | Inf | N/A |
| UBR-CG-C | NPT1-CM-W | 0.281 | 0 | Inf | N/A |
| UBR-CG-C | NPT2-CM-W | 0.281 | 0 | Inf | N/A |
| UBR-CG-C | NST1-CM-W | 0.378 | 0 | Inf | N/A |
| UBR-CG-C | NST2-CM-W | 0.121 | 0 | Inf | N/A |
| UBR-CG-C | STN-CM-C | 0.126 | 0 | Inf | N/A |
| UBR-CG-C | KSN2-CG-C | 0.207 | 0 | Inf | N/A |
| SBR-CG-C | SBR-CM-C | –0.017 | 0 | -Inf | N/A |
| SBR-CG-C | KSN1-CG-C | –0.274 | 0 | -Inf | N/A |
| SBR-CG-C | NYK-CG-C | –0.165 | 0 | -Inf | N/A |
| SBR-CG-C | SNK4-CM-C | –0.180 | 0 | -Inf | N/A |
| SBR-CG-C | SNK2-CM-W | –0.070 | 0 | -Inf | N/A |
| SBR-CG-C | SNK1-CM-W | –0.123 | 0 | -Inf | N/A |
| SBR-CG-C | SPB1-CM-W | –0.022 | 0 | -Inf | N/A |
| SBR-CG-C | SPB2-CM-W | –0.046 | 0 | -Inf | N/A |
| SBR-CG-C | NPT1-CM-W | 0.023 | 0 | Inf | N/A |
| SBR-CG-C | NPT2-CM-W | 0.023 | 0 | Inf | N/A |
| SBR-CG-C | NST1-CM-W | 0.120 | 0 | Inf | N/A |
| SBR-CG-C | NST2-CM-W | –0.137 | 0 | -Inf | N/A |
| SBR-CG-C | STN-CM-C | –0.132 | 0 | -Inf | N/A |
| SBR-CG-C | KSN2-CG-C | –0.051 | 0 | -Inf | N/A |
| SBR-CM-C | KSN1-CG-C | –0.257 | 0 | -Inf | N/A |
| SBR-CM-C | NYK-CG-C | –0.148 | 0 | -Inf | N/A |
| SBR-CM-C | SNK4-CM-C | –0.163 | 0 | -Inf | N/A |
| SBR-CM-C | SNK2-CM-W | –0.053 | 0 | -Inf | N/A |
| SBR-CM-C | SNK1-CM-W | –0.106 | 0 | -Inf | N/A |
| SBR-CM-C | SPB1-CM-W | –0.005 | 0 | -Inf | N/A |
| SBR-CM-C | SPB2-CM-W | –0.029 | 0 | -Inf | N/A |
| SBR-CM-C | NPT1-CM-W | 0.040 | 0 | Inf | N/A |
| SBR-CM-C | NPT2-CM-W | 0.040 | 0 | Inf | N/A |
| SBR-CM-C | NST1-CM-W | 0.137 | 0 | Inf | N/A |
| SBR-CM-C | NST2-CM-W | –0.120 | 0 | -Inf | N/A |
| SBR-CM-C | STN-CM-C | –0.115 | 0 | -Inf | N/A |
| SBR-CM-C | KSN2-CG-C | –0.034 | 0 | -Inf | N/A |
| KSN1-CG-C | NYK-CG-C | 0.109 | 0 | Inf | N/A |
| KSN1-CG-C | SNK4-CM-C | 0.094 | 0 | Inf | N/A |
| KSN1-CG-C | SNK2-CM-W | 0.204 | 0 | Inf | N/A |
| KSN1-CG-C | SNK1-CM-W | 0.151 | 0 | Inf | N/A |
| KSN1-CG-C | SPB1-CM-W | 0.252 | 0 | Inf | N/A |
| KSN1-CG-C | SPB2-CM-W | 0.228 | 0 | Inf | N/A |
| KSN1-CG-C | NPT1-CM-W | 0.297 | 0 | Inf | N/A |
| KSN1-CG-C | NPT2-CM-W | 0.297 | 0 | Inf | N/A |
| KSN1-CG-C | NST1-CM-W | 0.394 | 0 | Inf | N/A |
| KSN1-CG-C | NST2-CM-W | 0.137 | 0 | Inf | N/A |
| KSN1-CG-C | STN-CM-C | 0.142 | 0 | Inf | N/A |
| KSN1-CG-C | KSN2-CG-C | 0.223 | 0 | Inf | N/A |
| NYK-CG-C | SNK4-CM-C | –0.015 | 0 | -Inf | N/A |
| NYK-CG-C | SNK2-CM-W | 0.095 | 0 | Inf | N/A |
| NYK-CG-C | SNK1-CM-W | 0.042 | 0 | Inf | N/A |
| NYK-CG-C | SPB1-CM-W | 0.143 | 0 | Inf | N/A |
| NYK-CG-C | SPB2-CM-W | 0.119 | 0 | Inf | N/A |
| NYK-CG-C | NPT1-CM-W | 0.188 | 0 | Inf | N/A |
| NYK-CG-C | NPT2-CM-W | 0.188 | 0 | Inf | N/A |
| NYK-CG-C | NST1-CM-W | 0.285 | 0 | Inf | N/A |
| NYK-CG-C | NST2-CM-W | 0.028 | 0 | Inf | N/A |
| NYK-CG-C | STN-CM-C | 0.033 | 0 | Inf | N/A |
| NYK-CG-C | KSN2-CG-C | 0.114 | 0 | Inf | N/A |
| SNK4-CM-C | SNK2-CM-W | 0.110 | 0 | Inf | N/A |
| SNK4-CM-C | SNK1-CM-W | 0.057 | 0 | Inf | N/A |
| SNK4-CM-C | SPB1-CM-W | 0.158 | 0 | Inf | N/A |
| SNK4-CM-C | SPB2-CM-W | 0.134 | 0 | Inf | N/A |
| SNK4-CM-C | NPT1-CM-W | 0.203 | 0 | Inf | N/A |
| SNK4-CM-C | NPT2-CM-W | 0.203 | 0 | Inf | N/A |
| SNK4-CM-C | NST1-CM-W | 0.300 | 0 | Inf | N/A |
| SNK4-CM-C | NST2-CM-W | 0.043 | 0 | Inf | N/A |
| SNK4-CM-C | STN-CM-C | 0.048 | 0 | Inf | N/A |
| SNK4-CM-C | KSN2-CG-C | 0.129 | 0 | Inf | N/A |
| SNK2-CM-W | SNK1-CM-W | –0.053 | 0 | -Inf | N/A |
| SNK2-CM-W | SPB1-CM-W | 0.048 | 0 | Inf | N/A |
| SNK2-CM-W | SPB2-CM-W | 0.024 | 0 | Inf | N/A |
| SNK2-CM-W | NPT1-CM-W | 0.093 | 0 | Inf | N/A |
| SNK2-CM-W | NPT2-CM-W | 0.093 | 0 | Inf | N/A |
| SNK2-CM-W | NST1-CM-W | 0.190 | 0 | Inf | N/A |
| SNK2-CM-W | NST2-CM-W | –0.067 | 0 | -Inf | N/A |
| SNK2-CM-W | STN-CM-C | –0.062 | 0 | -Inf | N/A |
| SNK2-CM-W | KSN2-CG-C | 0.019 | 0 | Inf | N/A |
| SNK1-CM-W | SPB1-CM-W | 0.101 | 0 | Inf | N/A |
| SNK1-CM-W | SPB2-CM-W | 0.077 | 0 | Inf | N/A |
| SNK1-CM-W | NPT1-CM-W | 0.146 | 0 | Inf | N/A |
| SNK1-CM-W | NPT2-CM-W | 0.146 | 0 | Inf | N/A |
| SNK1-CM-W | NST1-CM-W | 0.243 | 0 | Inf | N/A |
| SNK1-CM-W | NST2-CM-W | –0.014 | 0 | -Inf | N/A |
| SNK1-CM-W | STN-CM-C | –0.009 | 0 | -Inf | N/A |
| SNK1-CM-W | KSN2-CG-C | 0.072 | 0 | Inf | N/A |
| SPB1-CM-W | SPB2-CM-W | –0.024 | 0 | -Inf | N/A |
| SPB1-CM-W | NPT1-CM-W | 0.045 | 0 | Inf | N/A |
| SPB1-CM-W | NPT2-CM-W | 0.045 | 0 | Inf | N/A |
| SPB1-CM-W | NST1-CM-W | 0.142 | 0 | Inf | N/A |
| SPB1-CM-W | NST2-CM-W | –0.115 | 0 | -Inf | N/A |
| SPB1-CM-W | STN-CM-C | –0.110 | 0 | -Inf | N/A |
| SPB1-CM-W | KSN2-CG-C | –0.029 | 0 | -Inf | N/A |
| SPB2-CM-W | NPT1-CM-W | 0.069 | 0 | Inf | N/A |
| SPB2-CM-W | NPT2-CM-W | 0.069 | 0 | Inf | N/A |
| SPB2-CM-W | NST1-CM-W | 0.166 | 0 | Inf | N/A |
| SPB2-CM-W | NST2-CM-W | –0.091 | 0 | -Inf | N/A |
| SPB2-CM-W | STN-CM-C | –0.086 | 0 | -Inf | N/A |
| SPB2-CM-W | KSN2-CG-C | –0.005 | 0 | -Inf | N/A |
| NPT1-CM-W | NPT2-CM-W | 0.000 | 0 | NaN | N/A |
| NPT1-CM-W | NST1-CM-W | 0.097 | 0 | Inf | N/A |
| NPT1-CM-W | NST2-CM-W | –0.160 | 0 | -Inf | N/A |
| NPT1-CM-W | STN-CM-C | –0.155 | 0 | -Inf | N/A |
| NPT1-CM-W | KSN2-CG-C | –0.074 | 0 | -Inf | N/A |
| NPT2-CM-W | NST1-CM-W | 0.097 | 0 | Inf | N/A |
| NPT2-CM-W | NST2-CM-W | –0.160 | 0 | -Inf | N/A |
| NPT2-CM-W | STN-CM-C | –0.155 | 0 | -Inf | N/A |
| NPT2-CM-W | KSN2-CG-C | –0.074 | 0 | -Inf | N/A |
| NST1-CM-W | NST2-CM-W | –0.257 | 0 | -Inf | N/A |
| NST1-CM-W | STN-CM-C | –0.252 | 0 | -Inf | N/A |
| NST1-CM-W | KSN2-CG-C | –0.171 | 0 | -Inf | N/A |
| NST2-CM-W | STN-CM-C | 0.005 | 0 | Inf | N/A |
| NST2-CM-W | KSN2-CG-C | 0.086 | 0 | Inf | N/A |
| STN-CM-C | KSN2-CG-C | 0.081 | 0 | Inf | N/A |

Inf, infinitive; N/A, not applicable

**Table S9** Comparison of observed heterozygosity (*H*_o_) for the *Mstnb* gene between catfish population

| **Population 1** | **Population 2** | **Difference of means** | **Standard**  **Error** | ***t*-test** | ***p*-value** |
| --- | --- | --- | --- | --- | --- |
| SNK3-CM-W | UBR-CM-C | 0.458 | 0 | Inf | N/A |
| SNK3-CM-W | UBR-CB-C | 0.708 | 0 | Inf | N/A |
| SNK3-CM-W | UBR-CG-C | –0.042 | 0 | -Inf | N/A |
| SNK3-CM-W | SBR-CG-C | 0.208 | 0 | Inf | N/A |
| SNK3-CM-W | SBR-CM-C | 0.152 | 0 | Inf | N/A |
| SNK3-CM-W | KSN1-CG-C | 0.082 | 0 | Inf | N/A |
| SNK3-CM-W | NYK-CG-C | 0.031 | 0 | Inf | N/A |
| SNK3-CM-W | SNK4-CM-C | 0.189 | 0 | Inf | N/A |
| SNK3-CM-W | SNK2-CM-W | 0.301 | 0 | Inf | N/A |
| SNK3-CM-W | SNK1-CM-W | 0.168 | 0 | Inf | N/A |
| SNK3-CM-W | SPB1-CM-W | 0.508 | 0 | Inf | N/A |
| SNK3-CM-W | SPB2-CM-W | 0.041 | 0 | Inf | N/A |
| SNK3-CM-W | NPT1-CM-W | 0.208 | 0 | Inf | N/A |
| SNK3-CM-W | NPT2-CM-W | 0.208 | 0 | Inf | N/A |
| SNK3-CM-W | NST1-CM-W | 0.375 | 0 | Inf | N/A |
| SNK3-CM-W | NST2-CM-W | 0.108 | 0 | Inf | N/A |
| SNK3-CM-W | STN-CM-C | 0.131 | 0 | Inf | N/A |
| SNK3-CM-W | KSN2-CG-C | 0.219 | 0 | Inf | N/A |
| UBR-CM-C | UBR-CB-C | 0.250 | 0 | Inf | N/A |
| UBR-CM-C | UBR-CG-C | –0.500 | 0 | -Inf | N/A |
| UBR-CM-C | SBR-CG-C | –0.250 | 0 | -Inf | N/A |
| UBR-CM-C | SBR-CM-C | –0.306 | 0 | -Inf | N/A |
| UBR-CM-C | KSN1-CG-C | –0.376 | 0 | -Inf | N/A |
| UBR-CM-C | NYK-CG-C | –0.427 | 0 | -Inf | N/A |
| UBR-CM-C | SNK4-CM-C | –0.269 | 0 | -Inf | N/A |
| UBR-CM-C | SNK2-CM-W | –0.157 | 0 | -Inf | N/A |
| UBR-CM-C | SNK1-CM-W | –0.290 | 0 | -Inf | N/A |
| UBR-CM-C | SPB1-CM-W | 0.050 | 0 | Inf | N/A |
| UBR-CM-C | SPB2-CM-W | –0.417 | 0 | -Inf | N/A |
| UBR-CM-C | NPT1-CM-W | –0.250 | 0 | -Inf | N/A |
| UBR-CM-C | NPT2-CM-W | –0.250 | 0 | -Inf | N/A |
| UBR-CM-C | NST1-CM-W | –0.083 | 0 | -Inf | N/A |
| UBR-CM-C | NST2-CM-W | –0.350 | 0 | -Inf | N/A |
| UBR-CM-C | STN-CM-C | –0.327 | 0 | -Inf | N/A |
| UBR-CM-C | KSN2-CG-C | –0.239 | 0 | -Inf | N/A |
| UBR-CB-C | UBR-CG-C | –0.750 | 0 | -Inf | N/A |
| UBR-CB-C | SBR-CG-C | –0.500 | 0 | -Inf | N/A |
| UBR-CB-C | SBR-CM-C | –0.556 | 0 | -Inf | N/A |
| UBR-CB-C | KSN1-CG-C | –0.626 | 0 | -Inf | N/A |
| UBR-CB-C | NYK-CG-C | –0.677 | 0 | -Inf | N/A |
| UBR-CB-C | SNK4-CM-C | –0.519 | 0 | -Inf | N/A |
| UBR-CB-C | SNK2-CM-W | –0.407 | 0 | -Inf | N/A |
| UBR-CB-C | SNK1-CM-W | –0.540 | 0 | -Inf | N/A |
| UBR-CB-C | SPB1-CM-W | –0.200 | 0 | -Inf | N/A |
| UBR-CB-C | SPB2-CM-W | –0.667 | 0 | -Inf | N/A |
| UBR-CB-C | NPT1-CM-W | –0.500 | 0 | -Inf | N/A |
| UBR-CB-C | NPT2-CM-W | –0.500 | 0 | -Inf | N/A |
| UBR-CB-C | NST1-CM-W | –0.333 | 0 | -Inf | N/A |
| UBR-CB-C | NST2-CM-W | –0.600 | 0 | -Inf | N/A |
| UBR-CB-C | STN-CM-C | –0.577 | 0 | -Inf | N/A |
| UBR-CB-C | KSN2-CG-C | –0.489 | 0 | -Inf | N/A |
| UBR-CG-C | SBR-CG-C | 0.250 | 0 | Inf | N/A |
| UBR-CG-C | SBR-CM-C | 0.194 | 0 | Inf | N/A |
| UBR-CG-C | KSN1-CG-C | 0.124 | 0 | Inf | N/A |
| UBR-CG-C | NYK-CG-C | 0.073 | 0 | Inf | N/A |
| UBR-CG-C | SNK4-CM-C | 0.231 | 0 | Inf | N/A |
| UBR-CG-C | SNK2-CM-W | 0.343 | 0 | Inf | N/A |
| UBR-CG-C | SNK1-CM-W | 0.210 | 0 | Inf | N/A |
| UBR-CG-C | SPB1-CM-W | 0.550 | 0 | Inf | N/A |
| UBR-CG-C | SPB2-CM-W | 0.083 | 0 | Inf | N/A |
| UBR-CG-C | NPT1-CM-W | 0.250 | 0 | Inf | N/A |
| UBR-CG-C | NPT2-CM-W | 0.250 | 0 | Inf | N/A |
| UBR-CG-C | NST1-CM-W | 0.417 | 0 | Inf | N/A |
| UBR-CG-C | NST2-CM-W | 0.150 | 0 | Inf | N/A |
| UBR-CG-C | STN-CM-C | 0.173 | 0 | Inf | N/A |
| UBR-CG-C | KSN2-CG-C | 0.261 | 0 | Inf | N/A |
| SBR-CG-C | SBR-CM-C | –0.056 | 0 | -Inf | N/A |
| SBR-CG-C | KSN1-CG-C | –0.126 | 0 | -Inf | N/A |
| SBR-CG-C | NYK-CG-C | –0.177 | 0 | -Inf | N/A |
| SBR-CG-C | SNK4-CM-C | –0.019 | 0 | -Inf | N/A |
| SBR-CG-C | SNK2-CM-W | 0.093 | 0 | Inf | N/A |
| SBR-CG-C | SNK1-CM-W | –0.040 | 0 | -Inf | N/A |
| SBR-CG-C | SPB1-CM-W | 0.300 | 0 | Inf | N/A |
| SBR-CG-C | SPB2-CM-W | –0.167 | 0 | -Inf | N/A |
| SBR-CG-C | NPT1-CM-W | 0.000 | 0 | NaN | N/A |
| SBR-CG-C | NPT2-CM-W | 0.000 | 0 | NaN | N/A |
| SBR-CG-C | NST1-CM-W | 0.167 | 0 | Inf | N/A |
| SBR-CG-C | NST2-CM-W | –0.100 | 0 | -Inf | N/A |
| SBR-CG-C | STN-CM-C | –0.077 | 0 | -Inf | N/A |
| SBR-CG-C | KSN2-CG-C | 0.011 | 0 | Inf | N/A |
| SBR-CM-C | KSN1-CG-C | –0.070 | 0 | -Inf | N/A |
| SBR-CM-C | NYK-CG-C | –0.121 | 0 | -Inf | N/A |
| SBR-CM-C | SNK4-CM-C | 0.037 | 0 | Inf | N/A |
| SBR-CM-C | SNK2-CM-W | 0.149 | 0 | Inf | N/A |
| SBR-CM-C | SNK1-CM-W | 0.016 | 0 | Inf | N/A |
| SBR-CM-C | SPB1-CM-W | 0.356 | 0 | Inf | N/A |
| SBR-CM-C | SPB2-CM-W | –0.111 | 0 | -Inf | N/A |
| SBR-CM-C | NPT1-CM-W | 0.056 | 0 | Inf | N/A |
| SBR-CM-C | NPT2-CM-W | 0.056 | 0 | Inf | N/A |
| SBR-CM-C | NST1-CM-W | 0.223 | 0 | Inf | N/A |
| SBR-CM-C | NST2-CM-W | –0.044 | 0 | -Inf | N/A |
| SBR-CM-C | STN-CM-C | –0.021 | 0 | -Inf | N/A |
| SBR-CM-C | KSN2-CG-C | 0.067 | 0 | Inf | N/A |
| KSN1-CG-C | NYK-CG-C | –0.051 | 0 | -Inf | N/A |
| KSN1-CG-C | SNK4-CM-C | 0.107 | 0 | Inf | N/A |
| KSN1-CG-C | SNK2-CM-W | 0.219 | 0 | Inf | N/A |
| KSN1-CG-C | SNK1-CM-W | 0.086 | 0 | Inf | N/A |
| KSN1-CG-C | SPB1-CM-W | 0.426 | 0 | Inf | N/A |
| KSN1-CG-C | SPB2-CM-W | –0.041 | 0 | -Inf | N/A |
| KSN1-CG-C | NPT1-CM-W | 0.126 | 0 | Inf | N/A |
| KSN1-CG-C | NPT2-CM-W | 0.126 | 0 | Inf | N/A |
| KSN1-CG-C | NST1-CM-W | 0.293 | 0 | Inf | N/A |
| KSN1-CG-C | NST2-CM-W | 0.026 | 0 | Inf | N/A |
| KSN1-CG-C | STN-CM-C | 0.049 | 0 | Inf | N/A |
| KSN1-CG-C | KSN2-CG-C | 0.137 | 0 | Inf | N/A |
| NYK-CG-C | SNK4-CM-C | 0.158 | 0 | Inf | N/A |
| NYK-CG-C | SNK2-CM-W | 0.270 | 0 | Inf | N/A |
| NYK-CG-C | SNK1-CM-W | 0.137 | 0 | Inf | N/A |
| NYK-CG-C | SPB1-CM-W | 0.477 | 0 | Inf | N/A |
| NYK-CG-C | SPB2-CM-W | 0.010 | 0 | Inf | N/A |
| NYK-CG-C | NPT1-CM-W | 0.177 | 0 | Inf | N/A |
| NYK-CG-C | NPT2-CM-W | 0.177 | 0 | Inf | N/A |
| NYK-CG-C | NST1-CM-W | 0.344 | 0 | Inf | N/A |
| NYK-CG-C | NST2-CM-W | 0.077 | 0 | Inf | N/A |
| NYK-CG-C | STN-CM-C | 0.100 | 0 | Inf | N/A |
| NYK-CG-C | KSN2-CG-C | 0.188 | 0 | Inf | N/A |
| SNK4-CM-C | SNK2-CM-W | 0.112 | 0 | Inf | N/A |
| SNK4-CM-C | SNK1-CM-W | –0.021 | 0 | -Inf | N/A |
| SNK4-CM-C | SPB1-CM-W | 0.319 | 0 | Inf | N/A |
| SNK4-CM-C | SPB2-CM-W | –0.148 | 0 | -Inf | N/A |
| SNK4-CM-C | NPT1-CM-W | 0.019 | 0 | Inf | N/A |
| SNK4-CM-C | NPT2-CM-W | 0.019 | 0 | Inf | N/A |
| SNK4-CM-C | NST1-CM-W | 0.186 | 0 | Inf | N/A |
| SNK4-CM-C | NST2-CM-W | –0.081 | 0 | -Inf | N/A |
| SNK4-CM-C | STN-CM-C | –0.058 | 0 | -Inf | N/A |
| SNK4-CM-C | KSN2-CG-C | 0.030 | 0 | Inf | N/A |
| SNK2-CM-W | SNK1-CM-W | –0.133 | 0 | -Inf | N/A |
| SNK2-CM-W | SPB1-CM-W | 0.207 | 0 | Inf | N/A |
| SNK2-CM-W | SPB2-CM-W | –0.260 | 0 | -Inf | N/A |
| SNK2-CM-W | NPT1-CM-W | –0.093 | 0 | -Inf | N/A |
| SNK2-CM-W | NPT2-CM-W | –0.093 | 0 | -Inf | N/A |
| SNK2-CM-W | NST1-CM-W | 0.074 | 0 | Inf | N/A |
| SNK2-CM-W | NST2-CM-W | –0.193 | 0 | -Inf | N/A |
| SNK2-CM-W | STN-CM-C | –0.170 | 0 | -Inf | N/A |
| SNK2-CM-W | KSN2-CG-C | –0.082 | 0 | -Inf | N/A |
| SNK1-CM-W | SPB1-CM-W | 0.340 | 0 | Inf | N/A |
| SNK1-CM-W | SPB2-CM-W | –0.127 | 0 | -Inf | N/A |
| SNK1-CM-W | NPT1-CM-W | 0.040 | 0 | Inf | N/A |
| SNK1-CM-W | NPT2-CM-W | 0.040 | 0 | Inf | N/A |
| SNK1-CM-W | NST1-CM-W | 0.207 | 0 | Inf | N/A |
| SNK1-CM-W | NST2-CM-W | –0.060 | 0 | -Inf | N/A |
| SNK1-CM-W | STN-CM-C | –0.037 | 0 | -Inf | N/A |
| SNK1-CM-W | KSN2-CG-C | 0.051 | 0 | Inf | N/A |
| SPB1-CM-W | SPB2-CM-W | –0.467 | 0 | -Inf | N/A |
| SPB1-CM-W | NPT1-CM-W | –0.300 | 0 | -Inf | N/A |
| SPB1-CM-W | NPT2-CM-W | –0.300 | 0 | -Inf | N/A |
| SPB1-CM-W | NST1-CM-W | –0.133 | 0 | -Inf | N/A |
| SPB1-CM-W | NST2-CM-W | –0.400 | 0 | -Inf | N/A |
| SPB1-CM-W | STN-CM-C | –0.377 | 0 | -Inf | N/A |
| SPB1-CM-W | KSN2-CG-C | –0.289 | 0 | -Inf | N/A |
| SPB2-CM-W | NPT1-CM-W | 0.167 | 0 | Inf | N/A |
| SPB2-CM-W | NPT2-CM-W | 0.167 | 0 | Inf | N/A |
| SPB2-CM-W | NST1-CM-W | 0.334 | 0 | Inf | N/A |
| SPB2-CM-W | NST2-CM-W | 0.067 | 0 | Inf | N/A |
| SPB2-CM-W | STN-CM-C | 0.090 | 0 | Inf | N/A |
| SPB2-CM-W | KSN2-CG-C | 0.178 | 0 | Inf | N/A |
| NPT1-CM-W | NPT2-CM-W | 0.000 | 0 | NaN | N/A |
| NPT1-CM-W | NST1-CM-W | 0.167 | 0 | Inf | N/A |
| NPT1-CM-W | NST2-CM-W | –0.100 | 0 | -Inf | N/A |
| NPT1-CM-W | STN-CM-C | –0.077 | 0 | -Inf | N/A |
| NPT1-CM-W | KSN2-CG-C | 0.011 | 0 | Inf | N/A |
| NPT2-CM-W | NST1-CM-W | 0.167 | 0 | Inf | N/A |
| NPT2-CM-W | NST2-CM-W | –0.100 | 0 | -Inf | N/A |
| NPT2-CM-W | STN-CM-C | –0.077 | 0 | -Inf | N/A |
| NPT2-CM-W | KSN2-CG-C | 0.011 | 0 | Inf | N/A |
| NST1-CM-W | NST2-CM-W | –0.267 | 0 | -Inf | N/A |
| NST1-CM-W | STN-CM-C | –0.244 | 0 | -Inf | N/A |
| NST1-CM-W | KSN2-CG-C | –0.156 | 0 | -Inf | N/A |
| NST2-CM-W | STN-CM-C | 0.023 | 0 | Inf | N/A |
| NST2-CM-W | KSN2-CG-C | 0.111 | 0 | Inf | N/A |
| STN-CM-C | KSN2-CG-C | 0.088 | 0 | Inf | N/A |

Inf, infinitive; N/A, not applicable

**Table S10** Detailed site-by-site results from the MEME analysis

| **Codon** | **α^1^** | **β1^2^** | **p1^3^** | **β+^4^** | **p+^5^** | **LRT^6^** | ***p*-value^7^** | **q^8^** | **Class^9^** |
| --- | --- | --- | --- | --- | --- | --- | --- | --- | --- |
| 1 | 0.000 | 0.000 | 1.000 | 0.000 | 0 | 0.000 | 1.000 | 1 | Invariable |
| 2 | 0.000 | 0.000 | 1.000 | 0.000 | 0 | 0.000 | 1.000 | 1 | Invariable |
| 3 | 0.000 | 0.000 | 0.000 | 58.644 | 1 | 5.992 | 0.023 | 1 | Diversifying |
| 4 | 0.000 | 0.000 | 1.000 | 0.000 | 0 | 0.000 | 1.000 | 1 | Invariable |
| 5 | 0.000 | 0.000 | 1.000 | 0.000 | 0 | 0.000 | 1.000 | 1 | Invariable |
| 6 | 0.001 | 0.000 | 0.000 | 6.663 | 1 | 3.467 | 0.084 | 1 | Neutral |
| 7 | 0.000 | 0.000 | 1.000 | 0.000 | 0 | 0.000 | 1.000 | 1 | Invariable |
| 8 | 0.000 | 0.000 | 1.000 | 0.000 | 0 | 0.000 | 1.000 | 1 | Invariable |
| 9 | 0.000 | 0.000 | 1.000 | 0.000 | 0 | 0.000 | 1.000 | 1 | Invariable |
| 10 | 0.000 | 0.000 | 1.000 | 0.000 | 0 | 0.000 | 1.000 | 1 | Invariable |
| 11 | 7.543 | 0.808 | 0.000 | 0.605 | 1 | 0.000 | 0.667 | 1 | Neutral |
| 12 | 0.000 | 0.000 | 0.000 | 0.967 | 1 | 0.931 | 0.331 | 1 | Neutral |
| 13 | 0.000 | 0.000 | 0.000 | 2.183 | 1 | 2.037 | 0.178 | 1 | Neutral |
| 14 | 2.107 | 0.000 | 0.000 | 0.769 | 1 | 0.000 | 0.667 | 1 | Neutral |
| 15 | 2.169 | 0.875 | 0.000 | 0.924 | 1 | 0.000 | 0.667 | 1 | Neutral |
| 16 | 3.139 | 3.139 | 0.000 | 3.095 | 1 | 0.000 | 0.667 | 1 | Neutral |
| 17 | 0.000 | 0.000 | 1.000 | 0.000 | 0 | 0.000 | 1.000 | 1 | Invariable |
| 18 | 1.483 | 0.124 | 0.000 | 1.649 | 1 | -0.001 | 0.667 | 1 | Neutral |
| 19 | 2.534 | 1.667 | 0.000 | 1.684 | 1 | 0.000 | 0.667 | 1 | Neutral |
| 20 | 0.000 | 0.000 | 0.000 | 4.389 | 1 | 2.595 | 0.132 | 1 | Neutral |
| 21 | 2.305 | 0.496 | 0.000 | 0.539 | 1 | 0.000 | 0.667 | 1 | Neutral |
| 22 | 2.528 | 1.851 | 0.000 | 36.656 | 1 | 0.839 | 0.349 | 1 | Neutral |
| 23 | 2.907 | 0.602 | 0.000 | 0.634 | 1 | 0.000 | 0.667 | 1 | Neutral |
| 24 | 0.000 | 0.000 | 0.000 | 2.256 | 1 | 2.306 | 0.154 | 1 | Neutral |
| 25 | 1.013 | 0.022 | 0.000 | 3.320 | 1 | 0.165 | 0.543 | 1 | Neutral |
| 26 | 1.889 | 0.991 | 0.000 | 286.315 | 1 | 3.354 | 0.089 | 1 | Neutral |
| 27 | 2.231 | 1.854 | 0.000 | 1.718 | 1 | 0.000 | 0.667 | 1 | Neutral |
| 28 | 0.000 | 0.000 | 0.000 | 0.601 | 1 | 0.613 | 0.400 | 1 | Neutral |
| 29 | 2.145 | 0.109 | 0.000 | 3.408 | 1 | 0.029 | 0.621 | 1 | Neutral |
| 30 | 2.384 | 0.663 | 0.000 | 7.858 | 1 | 0.173 | 0.540 | 1 | Neutral |
| 31 | 34.471 | 2.801 | 0.000 | 5.255 | 1 | 0.000 | 0.667 | 1 | Neutral |
| 32 | 0.884 | 0.009 | 0.000 | 3.442 | 1 | 0.175 | 0.539 | 1 | Neutral |
| 33 | 1.312 | 0.014 | 0.000 | 2.930 | 1 | 0.067 | 0.593 | 1 | Neutral |
| 34 | 0.000 | 0.000 | 0.000 | 2.147 | 1 | 2.414 | 0.146 | 1 | Neutral |
| 35 | 1.203 | 0.083 | 0.000 | 2.401 | 1 | 0.072 | 0.590 | 1 | Neutral |
| 36 | 4.271 | 0.814 | 0.000 | 0.893 | 1 | 0.000 | 0.667 | 1 | Neutral |
| 37 | 1.643 | 0.000 | 0.000 | 1.053 | 1 | 0.000 | 0.667 | 1 | Neutral |
| 38 | 1.265 | 0.063 | 0.000 | 1.343 | 1 | 0.000 | 0.667 | 1 | Neutral |
| 39 | 1.869 | 0.065 | 0.000 | 2.668 | 1 | 0.011 | 0.639 | 1 | Neutral |
| 40 | 10000.000 | 1.967 | 0.000 | 0.967 | 1 | 0.000 | 0.667 | 1 | Neutral |
| 41 | 0.000 | 0.000 | 0.000 | 4.965 | 1 | 1.329 | 0.263 | 1 | Neutral |
| 42 | 2.790 | 0.591 | 0.000 | 0.545 | 1 | 0.000 | 0.667 | 1 | Neutral |
| 43 | 2.325 | 1.851 | 0.000 | 1.921 | 1 | 0.000 | 0.667 | 1 | Neutral |
| 44 | 2.267 | 2.086 | 0.000 | 2.136 | 1 | 0.000 | 0.667 | 1 | Neutral |
| 45 | 0.000 | 0.000 | 0.000 | 2.294 | 1 | 2.306 | 0.154 | 1 | Neutral |
| 46 | 1.999 | 0.018 | 0.000 | 3.354 | 1 | 0.041 | 0.611 | 1 | Neutral |
| 47 | 0.000 | 0.000 | 0.000 | 2.929 | 1 | 2.358 | 0.150 | 1 | Neutral |
| 48 | 0.000 | 0.000 | 0.000 | 0.905 | 1 | 1.114 | 0.297 | 1 | Neutral |
| 49 | 3.157 | 0.000 | 0.000 | 0.465 | 1 | 0.000 | 0.667 | 1 | Neutral |
| 50 | 2.198 | 1.696 | 0.000 | 1.786 | 1 | 0.000 | 0.667 | 0.995 | Neutral |
| 51 | 3.196 | 2.043 | 0.000 | 1.993 | 1 | 0.000 | 0.667 | 0.98 | Neutral |
| 52 | 4.834 | 0.845 | 0.000 | 0.820 | 1 | 0.000 | 0.667 | 0.966 | Neutral |
| 53 | 1.446 | 1.037 | 0.000 | 1.132 | 1 | 0.000 | 0.667 | 0.952 | Neutral |
| 54 | 0.700 | 0.023 | 0.000 | 2.738 | 1 | 0.141 | 0.554 | 1 | Neutral |
| 55 | 0.000 | 0.000 | 0.000 | 0.936 | 1 | 0.312 | 0.487 | 1 | Neutral |
| 56 | 1.990 | 0.123 | 0.000 | 8.046 | 1 | 0.374 | 0.466 | 1 | Neutral |
| 57 | 2.341 | 1.348 | 0.000 | 1.315 | 1 | 0.000 | 0.667 | 0.939 | Neutral |
| 58 | 0.000 | 0.000 | 1.000 | 0.000 | 0 | 0.000 | 1.000 | 1 | Invariable |
| 59 | 4.432 | 2.191 | 0.000 | 2.008 | 1 | 0.000 | 0.667 | 0.926 | Neutral |
| 60 | 2.107 | 0.096 | 0.000 | 2.879 | 1 | 0.013 | 0.637 | 1 | Neutral |
| 61 | 0.002 | 0.000 | 0.000 | 148.314 | 1 | 1.899 | 0.192 | 1 | Neutral |
| 62 | 2.288 | 0.947 | 0.000 | 0.901 | 1 | 0.000 | 0.667 | 0.913 | Neutral |
| 63 | 1.974 | 1.416 | 0.000 | 1.524 | 1 | 0.000 | 0.667 | 0.901 | Neutral |
| 64 | 2.643 | 1.993 | 0.000 | 1.925 | 1 | 0.000 | 0.667 | 0.889 | Neutral |
| 65 | 0.000 | 0.000 | 0.000 | 5.589 | 1 | 2.345 | 0.151 | 1 | Neutral |
| 66 | 0.002 | 0.000 | 0.000 | 3.316 | 1 | 0.120 | 0.564 | 1 | Neutral |
| 67 | 2.208 | 0.023 | 0.000 | 2.207 | 1 | 0.000 | 0.667 | 0.877 | Neutral |
| 68 | 0.000 | 0.000 | 0.000 | 2.659 | 1 | 2.003 | 0.182 | 1 | Neutral |
| 69 | 2.556 | 0.572 | 0.000 | 0.611 | 1 | 0.000 | 0.667 | 0.866 | Neutral |
| 70 | 0.000 | 0.000 | 0.000 | 8.017 | 1 | 1.183 | 0.286 | 1 | Neutral |
| 71 | 0.000 | 0.000 | 0.000 | 1.060 | 1 | 1.261 | 0.274 | 1 | Neutral |
| 72 | 1.466 | 0.708 | 0.000 | 44.563 | 1 | 1.633 | 0.222 | 1 | Neutral |
| 73 | 1.446 | 1.037 | 0.000 | 1.132 | 1 | 0.000 | 0.667 | 0.855 | Neutral |
| 74 | 1.375 | 0.000 | 0.000 | 0.495 | 1 | 0.000 | 0.667 | 0.844 | Neutral |
| 75 | 0.000 | 0.000 | 1.000 | 0.000 | 0 | 0.000 | 1.000 | 1 | Invariable |
| 76 | 1.447 | 0.000 | 0.000 | 0.520 | 1 | 0.000 | 0.667 | 0.833 | Neutral |
| 77 | 1.515 | 0.127 | 0.000 | 1.567 | 1 | 0.001 | 0.659 | 1 | Neutral |
| 78 | 2.102 | 0.017 | 0.000 | 2.891 | 1 | 0.026 | 0.623 | 1 | Neutral |
| 79 | 1.991 | 0.023 | 0.000 | 3.084 | 1 | 0.030 | 0.620 | 1 | Neutral |
| 80 | 0.000 | 0.000 | 0.000 | 0.872 | 1 | 0.911 | 0.334 | 1 | Neutral |
| 81 | 2.867 | 2.525 | 0.000 | 2.392 | 1 | 0.000 | 0.667 | 0.823 | Neutral |
| 82 | 2.330 | 0.786 | 0.000 | 0.734 | 1 | 0.000 | 0.667 | 0.813 | Neutral |
| 83 | 0.000 | 0.000 | 0.000 | 1.161 | 1 | 1.314 | 0.266 | 1 | Neutral |
| 84 | 2.325 | 1.851 | 0.000 | 1.921 | 1 | 0.000 | 0.667 | 0.803 | Neutral |
| 85 | 2.198 | 1.677 | 0.000 | 1.735 | 1 | 0.000 | 0.667 | 0.794 | Neutral |
| 86 | 1.083 | 0.000 | 0.000 | 13.750 | 1 | 0.757 | 0.366 | 1 | Neutral |
| 87 | 3.257 | 2.541 | 0.000 | 2.664 | 1 | 0.000 | 0.667 | 0.784 | Neutral |
| 88 | 0.000 | 0.000 | 0.000 | 0.487 | 1 | 0.555 | 0.415 | 1 | Neutral |
| 89 | 3.461 | 0.569 | 0.000 | 84.246 | 1 | 0.455 | 0.442 | 1 | Neutral |
| 90 | 31.968 | 6.531 | 0.000 | 6.503 | 1 | 0.000 | 0.667 | 0.775 | Neutral |
| 91 | 0.000 | 0.000 | 0.000 | 5.237 | 1 | 2.448 | 0.143 | 1 | Neutral |
| 92 | 86.482 | 33.472 | 0.000 | 32.582 | 1 | 0.000 | 0.667 | 0.766 | Neutral |
| 93 | 14.923 | 0.953 | 0.000 | 30.406 | 1 | 0.129 | 0.560 | 1 | Neutral |
| 94 | 17.751 | 0.980 | 0.000 | 23.584 | 1 | 0.020 | 0.629 | 1 | Neutral |
| 95 | 0.017 | 0.000 | 0.000 | 59.077 | 1 | 1.200 | 0.283 | 1 | Neutral |
| 96 | 22.440 | 0.000 | 0.000 | 27.597 | 1 | 0.012 | 0.638 | 1 | Neutral |
| 97 | 0.000 | 0.000 | 0.000 | 21.225 | 1 | 1.306 | 0.267 | 1 | Neutral |
| 98 | 31.612 | 22.726 | 0.000 | 21.176 | 1 | 0.000 | 0.667 | 0.758 | Neutral |
| 99 | 28.407 | 21.207 | 0.000 | 22.095 | 1 | 0.000 | 0.667 | 0.749 | Neutral |
| 100 | 0.037 | 0.000 | 0.000 | 38.769 | 1 | 0.435 | 0.448 | 1 | Neutral |

^1^ synonymous substitution rate (α); ^2^ nonsynonymous substitution rate under negative selection (β1); ^3^ proportion of branches evolving under negative selection (p1); ^4^ nonsynonymous substitution rate under positive selection (β+); ^5^ proportions of branches evolving under positive selection (*p+*); ^6^ the likelihood ratio test statistic (LTR); ^7^ *p*-value represents the significance level for episodic diversifying selection; ^8^ the false discovery rate–adjusted *p*-value; ^9^ inferred selection regime at each codon (invariable, neutral, or diversifying).

**Table S11** Detailed site-by-site results from the FEL analysis

| **Codon** | **Alpha^1^** | **Beta^2^** | **Alpha=Beta^3^** | **LRT^4^** | ***p*-value^5^** | **Total branch length** | **Class^6^** |
| --- | --- | --- | --- | --- | --- | --- | --- |
| 1 | 0.000 | 0.000 | 0.000 | 0.000 | 1.000 | 0.000 | Invariable |
| 2 | 0.000 | 0.000 | 0.000 | 0.000 | 1.000 | 0.000 | Invariable |
| 3 | 0.000 | 3.309 | 4.053 | 2.454 | 0.117 | 5.837 | Neutral |
| 4 | 0.000 | 0.000 | 0.000 | 0.000 | 1.000 | 0.000 | Invariable |
| 5 | 0.000 | 0.000 | 0.000 | 0.000 | 1.000 | 0.000 | Invariable |
| 6 | 0.000 | 0.256 | 0.206 | 0.400 | 0.527 | 0.297 | Neutral |
| 7 | 0.000 | 0.000 | 0.000 | 0.000 | 1.000 | 0.000 | Invariable |
| 8 | 0.000 | 0.000 | 0.000 | 0.000 | 1.000 | 0.000 | Invariable |
| 9 | 0.000 | 0.000 | 0.000 | 0.000 | 1.000 | 0.000 | Invariable |
| 10 | 0.000 | 0.000 | 0.000 | 0.000 | 1.000 | 0.000 | Invariable |
| 11 | 5.562 | 0.577 | 1.584 | 0.437 | 0.509 | 2.281 | Neutral |
| 12 | 0.000 | 0.675 | 0.449 | 0.930 | 0.335 | 0.646 | Neutral |
| 13 | 0.000 | 1.563 | 1.011 | 2.038 | 0.153 | 1.456 | Neutral |
| 14 | 1.486 | 0.000 | 0.287 | 2.649 | 0.104 | 0.414 | Neutral |
| 15 | 1.553 | 0.618 | 0.955 | 0.105 | 0.746 | 1.376 | Neutral |
| 16 | 2.222 | 2.161 | 2.188 | 0.000 | 0.992 | 3.151 | Neutral |
| 17 | 0.000 | 0.000 | 0.000 | 0.000 | 1.000 | 0.000 | Invariable |
| 18 | 1.085 | 1.119 | 1.105 | 0.000 | 0.988 | 1.591 | Neutral |
| 19 | 1.851 | 1.183 | 1.514 | 0.048 | 0.827 | 2.180 | Neutral |
| 20 | 0.000 | 3.072 | 3.179 | 2.596 | 0.107 | 4.579 | Neutral |
| 21 | 1.594 | 0.353 | 0.652 | 0.707 | 0.401 | 0.939 | Neutral |
| 22 | 1.930 | 3.551 | 3.139 | 0.074 | 0.786 | 4.521 | Neutral |
| 23 | 2.036 | 0.399 | 0.800 | 0.959 | 0.327 | 1.152 | Neutral |
| 24 | 0.000 | 1.583 | 0.910 | 2.306 | 0.129 | 1.310 | Neutral |
| 25 | 0.714 | 2.376 | 1.664 | 0.164 | 0.685 | 2.396 | Neutral |
| 26 | 1.248 | 9.162 | 4.370 | 0.676 | 0.411 | 6.293 | Neutral |
| 27 | 1.585 | 1.333 | 1.425 | 0.009 | 0.924 | 2.053 | Neutral |
| 28 | 0.000 | 0.433 | 0.300 | 0.613 | 0.434 | 0.432 | Neutral |
| 29 | 1.620 | 2.274 | 2.074 | 0.035 | 0.852 | 2.988 | Neutral |
| 30 | 2.003 | 0.900 | 1.019 | 0.108 | 0.742 | 1.467 | Neutral |
| 31 | 25.559 | 2.048 | 4.143 | 2.181 | 0.140 | 5.967 | Neutral |
| 32 | 0.589 | 2.529 | 1.736 | 0.175 | 0.676 | 2.500 | Neutral |
| 33 | 0.924 | 2.119 | 1.743 | 0.067 | 0.795 | 2.510 | Neutral |
| 34 | 0.000 | 1.529 | 0.901 | 2.414 | 0.120 | 1.298 | Neutral |
| 35 | 0.864 | 1.695 | 1.326 | 0.072 | 0.789 | 1.910 | Neutral |
| 36 | 3.001 | 0.565 | 1.280 | 0.713 | 0.398 | 1.843 | Neutral |
| 37 | 1.171 | 0.000 | 0.316 | 2.122 | 0.145 | 0.456 | Neutral |
| 38 | 0.913 | 0.947 | 0.929 | 0.000 | 0.988 | 1.339 | Neutral |
| 39 | 1.392 | 1.842 | 1.734 | 0.012 | 0.913 | 2.498 | Neutral |
| 40 | 10000.000 | 1.278 | 1.560 | 0.135 | 0.713 | 2.246 | Neutral |
| 41 | 0.000 | 3.499 | 4.191 | 1.329 | 0.249 | 6.036 | Neutral |
| 42 | 2.075 | 0.425 | 0.761 | 0.651 | 0.420 | 1.096 | Neutral |
| 43 | 1.585 | 1.343 | 1.432 | 0.007 | 0.934 | 2.062 | Neutral |
| 44 | 1.615 | 1.500 | 1.534 | 0.001 | 0.971 | 2.210 | Neutral |
| 45 | 0.000 | 1.638 | 1.006 | 2.306 | 0.129 | 1.449 | Neutral |
| 46 | 1.468 | 2.278 | 2.061 | 0.041 | 0.839 | 2.968 | Neutral |
| 47 | 0.000 | 2.051 | 1.431 | 2.358 | 0.125 | 2.060 | Neutral |
| 48 | 0.000 | 0.647 | 0.330 | 1.113 | 0.291 | 0.475 | Neutral |
| 49 | 2.239 | 0.000 | 0.570 | 3.418 | 0.065 | 0.821 | Neutral |
| 50 | 1.598 | 1.214 | 1.350 | 0.022 | 0.881 | 1.945 | Neutral |
| 51 | 2.172 | 1.394 | 1.560 | 0.051 | 0.821 | 2.246 | Neutral |
| 52 | 3.508 | 0.584 | 1.172 | 0.605 | 0.437 | 1.688 | Neutral |
| 53 | 1.038 | 0.703 | 0.864 | 0.036 | 0.849 | 1.245 | Neutral |
| 54 | 0.465 | 1.917 | 1.415 | 0.141 | 0.708 | 2.037 | Neutral |
| 55 | 0.000 | 0.658 | 0.617 | 0.312 | 0.577 | 0.889 | Neutral |
| 56 | 1.259 | 5.827 | 3.773 | 0.376 | 0.540 | 5.433 | Neutral |
| 57 | 1.675 | 0.958 | 1.177 | 0.100 | 0.752 | 1.695 | Neutral |
| 58 | 0.000 | 0.000 | 0.000 | 0.000 | 1.000 | 0.000 | Invariable |
| 59 | 3.072 | 1.512 | 1.821 | 0.110 | 0.740 | 2.622 | Neutral |
| 60 | 1.533 | 1.976 | 1.836 | 0.014 | 0.907 | 2.644 | Neutral |
| 61 | 0.000 | 10.769 | 4.449 | 1.134 | 0.287 | 6.408 | Neutral |
| 62 | 1.697 | 0.671 | 0.972 | 0.227 | 0.634 | 1.399 | Neutral |
| 63 | 1.420 | 0.997 | 1.117 | 0.010 | 0.922 | 1.608 | Neutral |
| 64 | 1.874 | 1.400 | 1.491 | 0.008 | 0.930 | 2.147 | Neutral |
| 65 | 0.000 | 3.922 | 4.099 | 2.346 | 0.126 | 5.904 | Neutral |
| 66 | 0.000 | 2.348 | 2.071 | 0.119 | 0.730 | 2.983 | Neutral |
| 67 | 1.517 | 1.601 | 1.591 | 0.000 | 0.991 | 2.292 | Neutral |
| 68 | 0.000 | 1.870 | 1.414 | 2.004 | 0.157 | 2.036 | Neutral |
| 69 | 1.824 | 0.412 | 0.711 | 0.696 | 0.404 | 1.024 | Neutral |
| 70 | 0.000 | 5.752 | 3.810 | 1.183 | 0.277 | 5.488 | Neutral |
| 71 | 0.000 | 0.741 | 0.540 | 1.261 | 0.262 | 0.777 | Neutral |
| 72 | 0.894 | 6.191 | 3.181 | 0.702 | 0.402 | 4.581 | Neutral |
| 73 | 1.038 | 0.703 | 0.864 | 0.036 | 0.849 | 1.245 | Neutral |
| 74 | 0.970 | 0.000 | 0.280 | 2.189 | 0.139 | 0.403 | Neutral |
| 75 | 0.000 | 0.000 | 0.000 | 0.000 | 1.000 | 0.000 | Invariable |
| 76 | 1.034 | 0.000 | 0.283 | 2.232 | 0.135 | 0.408 | Neutral |
| 77 | 1.009 | 1.112 | 1.063 | 0.003 | 0.957 | 1.531 | Neutral |
| 78 | 1.414 | 2.112 | 1.877 | 0.027 | 0.868 | 2.703 | Neutral |
| 79 | 1.414 | 2.156 | 1.899 | 0.030 | 0.863 | 2.736 | Neutral |
| 80 | 0.000 | 0.623 | 0.343 | 0.911 | 0.340 | 0.494 | Neutral |
| 81 | 2.035 | 1.738 | 1.893 | 0.005 | 0.944 | 2.726 | Neutral |
| 82 | 1.663 | 0.534 | 0.888 | 0.361 | 0.548 | 1.279 | Neutral |
| 83 | 0.000 | 0.840 | 0.395 | 1.314 | 0.252 | 0.569 | Neutral |
| 84 | 1.585 | 1.343 | 1.432 | 0.007 | 0.934 | 2.062 | Neutral |
| 85 | 1.614 | 1.193 | 1.314 | 0.023 | 0.878 | 1.893 | Neutral |
| 86 | 0.803 | 9.457 | 4.492 | 0.758 | 0.384 | 6.469 | Neutral |
| 87 | 2.180 | 1.835 | 1.917 | 0.006 | 0.938 | 2.761 | Neutral |
| 88 | 0.000 | 0.338 | 0.252 | 0.556 | 0.456 | 0.363 | Neutral |
| 89 | 16.132 | 18.727 | 17.810 | 0.007 | 0.935 | 25.650 | Neutral |
| 90 | 22.747 | 4.599 | 8.007 | 0.903 | 0.342 | 11.532 | Neutral |
| 91 | 0.000 | 3.651 | 3.878 | 2.450 | 0.118 | 5.585 | Neutral |
| 92 | 61.496 | 22.862 | 30.697 | 0.516 | 0.473 | 44.209 | Neutral |
| 93 | 10.343 | 21.360 | 17.659 | 0.129 | 0.720 | 25.432 | Neutral |
| 94 | 12.388 | 16.757 | 15.233 | 0.020 | 0.888 | 21.938 | Neutral |
| 95 | 0.000 | 46.399 | 27.466 | 1.219 | 0.270 | 39.556 | Neutral |
| 96 | 15.673 | 19.538 | 18.463 | 0.013 | 0.911 | 26.590 | Neutral |
| 97 | 0.000 | 14.815 | 11.630 | 1.306 | 0.253 | 16.749 | Neutral |
| 98 | 21.505 | 15.848 | 16.977 | 0.019 | 0.889 | 24.450 | Neutral |
| 99 | 20.422 | 15.180 | 16.253 | 0.024 | 0.877 | 23.407 | Neutral |
| 100 | 0.000 | 19.862 | 18.649 | 0.244 | 0.622 | 26.858 | Neutral |

^1^ Synonymous substitution rate at the codon site (Alpha); ^2^ Nonsynonymous substitution rate at the codon site (Beta); ^3^ Difference between nonsynonymous and synonymous substitution rates (Beta−Alpha); ^4^ Likelihood ratio test statistic for selection at each codon (LRT); ^5^ Significance level for selection inferred by FEL (*p*-value); ^6^ Inferred selection regime at each codon (Class).

**Table S12** Codon sites under selection identified by FUBAR analysis

| **Codon** | **Partition** | **α^1^** | **Β^2^** | **β–α^3^** | **Prob[α>β]^4^** | **Prob[α<β]^5^** | **BayesFactor[α<β]^6^** |
| --- | --- | --- | --- | --- | --- | --- | --- |
| 1 | 1 | 1.597 | 0.230 | –1.367 | 0.709 | 0.217 | 0.284 |
| 2 | 1 | 0.678 | 0.246 | –0.431 | 0.604 | 0.299 | 0.439 |
| 3 | 1 | 0.815 | 2.171 | 1.356 | 0.255 | 0.693 | 2.324 |
| 4 | 1 | 1.765 | 0.265 | –1.500 | 0.688 | 0.240 | 0.324 |
| 5 | 1 | 0.720 | 0.412 | –0.308 | 0.516 | 0.401 | 0.688 |
| 6 | 1 | 1.256 | 0.577 | –0.679 | 0.479 | 0.456 | 0.860 |
| 7 | 1 | 1.613 | 0.225 | –1.388 | 0.713 | 0.212 | 0.277 |
| 8 | 1 | 0.716 | 0.407 | –0.309 | 0.522 | 0.395 | 0.671 |
| 9 | 1 | 1.617 | 0.256 | –1.361 | 0.694 | 0.233 | 0.312 |
| 10 | 1 | 0.569 | 0.336 | –0.233 | 0.522 | 0.384 | 0.641 |
| 11 | 1 | 2.019 | 0.888 | –1.131 | 0.459 | 0.484 | 0.966 |
| 12 | 1 | 1.189 | 0.650 | –0.539 | 0.371 | 0.568 | 1.354 |
| 13 | 1 | 0.732 | 1.034 | 0.302 | 0.216 | 0.734 | 2.838 |
| 14 | 1 | 1.595 | 0.425 | –1.170 | 0.758 | 0.200 | 0.257 |
| 15 | 1 | 1.270 | 0.903 | –0.367 | 0.493 | 0.446 | 0.826 |
| 16 | 1 | 1.643 | 1.322 | –0.321 | 0.394 | 0.538 | 1.195 |
| 17 | 1 | 0.715 | 0.342 | –0.373 | 0.548 | 0.365 | 0.590 |
| 18 | 1 | 1.363 | 0.956 | –0.407 | 0.474 | 0.459 | 0.871 |
| 19 | 1 | 1.322 | 1.028 | –0.294 | 0.480 | 0.456 | 0.860 |
| 20 | 1 | 0.737 | 1.648 | 0.911 | 0.171 | 0.785 | 3.756 |
| 21 | 1 | 1.427 | 0.734 | –0.693 | 0.586 | 0.352 | 0.557 |
| 22 | 1 | 2.484 | 2.323 | –0.161 | 0.327 | 0.607 | 1.586 |
| 23 | 1 | 1.567 | 0.754 | –0.813 | 0.602 | 0.337 | 0.522 |
| 24 | 1 | 0.722 | 0.842 | 0.120 | 0.261 | 0.689 | 2.280 |
| 25 | 1 | 1.047 | 1.313 | 0.266 | 0.338 | 0.596 | 1.519 |
| 26 | 1 | 1.543 | 6.579 | 5.036 | 0.243 | 0.703 | 2.437 |
| 27 | 1 | 1.408 | 1.197 | –0.212 | 0.410 | 0.521 | 1.117 |
| 28 | 1 | 1.296 | 0.630 | –0.666 | 0.391 | 0.548 | 1.247 |
| 29 | 1 | 1.527 | 1.652 | 0.125 | 0.358 | 0.574 | 1.382 |
| 30 | 1 | 2.255 | 1.200 | –1.055 | 0.427 | 0.513 | 1.084 |
| 31 | 1 | 10.560 | 1.154 | –9.406 | 0.678 | 0.276 | 0.391 |
| 32 | 1 | 1.209 | 1.507 | 0.298 | 0.336 | 0.601 | 1.547 |
| 33 | 1 | 1.421 | 1.214 | –0.207 | 0.368 | 0.567 | 1.348 |
| 34 | 1 | 0.634 | 1.058 | 0.424 | 0.200 | 0.753 | 3.130 |
| 35 | 1 | 1.045 | 1.090 | 0.045 | 0.384 | 0.555 | 1.281 |
| 36 | 1 | 2.366 | 0.739 | –1.628 | 0.592 | 0.349 | 0.552 |
| 37 | 1 | 1.407 | 0.425 | –0.982 | 0.751 | 0.203 | 0.262 |
| 38 | 1 | 0.946 | 0.929 | –0.017 | 0.457 | 0.475 | 0.930 |
| 39 | 1 | 2.708 | 1.005 | –1.703 | 0.420 | 0.520 | 1.113 |
| 40 | 1 | 5.333 | 0.859 | –4.474 | 0.509 | 0.436 | 0.796 |
| 41 | 1 | 2.079 | 1.742 | –0.337 | 0.225 | 0.725 | 2.713 |
| 42 | 1 | 2.063 | 0.673 | –1.390 | 0.590 | 0.350 | 0.553 |
| 43 | 1 | 1.370 | 1.325 | –0.045 | 0.397 | 0.536 | 1.190 |
| 44 | 1 | 1.447 | 0.980 | –0.467 | 0.414 | 0.519 | 1.108 |
| 45 | 1 | 0.680 | 1.025 | 0.345 | 0.205 | 0.747 | 3.030 |
| 46 | 1 | 1.412 | 1.405 | –0.007 | 0.352 | 0.582 | 1.433 |
| 47 | 1 | 0.661 | 1.132 | 0.471 | 0.196 | 0.756 | 3.193 |
| 48 | 1 | 0.732 | 0.754 | 0.022 | 0.304 | 0.639 | 1.819 |
| 49 | 1 | 1.765 | 0.543 | –1.222 | 0.754 | 0.203 | 0.263 |
| 50 | 1 | 1.506 | 1.302 | –0.205 | 0.424 | 0.507 | 1.058 |
| 51 | 1 | 2.860 | 0.908 | –1.952 | 0.480 | 0.455 | 0.859 |
| 52 | 1 | 3.120 | 0.769 | –2.351 | 0.548 | 0.395 | 0.671 |
| 53 | 1 | 1.356 | 0.993 | –0.364 | 0.487 | 0.447 | 0.832 |
| 54 | 1 | 1.110 | 1.227 | 0.117 | 0.331 | 0.608 | 1.595 |
| 55 | 1 | 1.826 | 0.710 | –1.116 | 0.426 | 0.514 | 1.088 |
| 56 | 1 | 1.465 | 2.447 | 0.982 | 0.299 | 0.644 | 1.861 |
| 57 | 1 | 1.406 | 1.034 | –0.373 | 0.450 | 0.482 | 0.956 |
| 58 | 1 | 1.629 | 0.179 | –1.449 | 0.750 | 0.173 | 0.215 |
| 59 | 1 | 3.073 | 0.906 | –2.167 | 0.464 | 0.475 | 0.932 |
| 60 | 1 | 1.451 | 1.214 | –0.237 | 0.379 | 0.553 | 1.272 |
| 61 | 1 | 1.354 | 7.841 | 6.487 | 0.149 | 0.806 | 4.273 |
| 62 | 1 | 1.490 | 0.813 | –0.678 | 0.536 | 0.399 | 0.682 |
| 63 | 1 | 1.295 | 0.944 | –0.351 | 0.427 | 0.513 | 1.083 |
| 64 | 1 | 2.652 | 0.904 | –1.747 | 0.460 | 0.482 | 0.957 |
| 65 | 1 | 0.835 | 3.530 | 2.695 | 0.184 | 0.772 | 3.479 |
| 66 | 1 | 2.394 | 1.159 | –1.236 | 0.327 | 0.616 | 1.646 |
| 67 | 1 | 2.504 | 0.917 | –1.587 | 0.399 | 0.541 | 1.212 |
| 68 | 1 | 0.764 | 1.204 | 0.441 | 0.212 | 0.738 | 2.890 |
| 69 | 1 | 2.475 | 0.615 | –1.860 | 0.633 | 0.308 | 0.458 |
| 70 | 1 | 1.473 | 3.594 | 2.121 | 0.211 | 0.741 | 2.935 |
| 71 | 1 | 1.030 | 0.695 | –0.334 | 0.313 | 0.630 | 1.750 |
| 72 | 1 | 1.297 | 3.811 | 2.514 | 0.263 | 0.680 | 2.183 |
| 73 | 1 | 1.356 | 0.993 | –0.364 | 0.487 | 0.447 | 0.832 |
| 74 | 1 | 1.181 | 0.381 | –0.800 | 0.759 | 0.194 | 0.248 |
| 75 | 1 | 0.715 | 0.342 | –0.373 | 0.548 | 0.365 | 0.590 |
| 76 | 1 | 1.402 | 0.383 | –1.019 | 0.764 | 0.190 | 0.241 |
| 77 | 1 | 1.146 | 0.941 | –0.206 | 0.463 | 0.469 | 0.907 |
| 78 | 1 | 1.426 | 1.414 | –0.012 | 0.359 | 0.576 | 1.396 |
| 79 | 1 | 1.314 | 1.364 | 0.050 | 0.358 | 0.576 | 1.397 |
| 80 | 1 | 0.779 | 0.745 | –0.033 | 0.329 | 0.614 | 1.632 |
| 81 | 1 | 1.489 | 1.354 | –0.135 | 0.419 | 0.516 | 1.095 |
| 82 | 1 | 2.003 | 0.708 | –1.295 | 0.581 | 0.356 | 0.568 |
| 83 | 1 | 0.676 | 0.774 | 0.098 | 0.290 | 0.654 | 1.943 |
| 84 | 1 | 1.370 | 1.325 | –0.045 | 0.397 | 0.536 | 1.190 |
| 85 | 1 | 1.434 | 0.930 | –0.504 | 0.429 | 0.503 | 1.041 |
| 86 | 1 | 1.377 | 7.578 | 6.201 | 0.129 | 0.828 | 4.958 |
| 87 | 1 | 2.271 | 1.059 | –1.212 | 0.395 | 0.543 | 1.221 |
| 88 | 1 | 0.716 | 0.551 | –0.166 | 0.400 | 0.534 | 1.179 |
| 89 | 1 | 5.392 | 17.451 | 12.060 | 0.177 | 0.759 | 3.246 |
| 90 | 1 | 9.381 | 1.944 | –7.437 | 0.632 | 0.315 | 0.472 |
| 91 | 1 | 0.835 | 2.433 | 1.598 | 0.122 | 0.839 | 5.354 |
| 92 | 1 | 25.073 | 15.183 | –9.890 | 0.595 | 0.243 | 0.330 |
| 93 | 1 | 4.387 | 17.473 | 13.085 | 0.115 | 0.828 | 4.960 |
| 94 | 1 | 5.943 | 14.755 | 8.811 | 0.192 | 0.751 | 3.106 |
| 95 | 1 | 3.444 | 25.064 | 21.620 | 0.053 | 0.914 | 10.923 |
| 96 | 1 | 4.063 | 14.243 | 10.180 | 0.108 | 0.841 | 5.456 |
| 97 | 1 | 2.336 | 8.880 | 6.545 | 0.086 | 0.875 | 7.219 |
| 98 | 1 | 6.389 | 14.246 | 7.856 | 0.182 | 0.755 | 3.166 |
| 99 | 1 | 4.818 | 12.995 | 8.177 | 0.132 | 0.812 | 4.441 |
| 100 | 1 | 4.097 | 10.781 | 6.684 | 0.127 | 0.825 | 4.843 |

^1^ Synonymous substitution rate at the codon site (α); ^2^ Nonsynonymous substitution rate at the codon site (β); ^3^ Difference between nonsynonymous and synonymous substitution rates (β−α); ^4^ Posterior probability of negative (purifying) selection (Prob[α>β]); ^5^ Posterior probability of positive (diversifying) selection (Prob[α<β]); ^6^ Bayes factor supporting positive selection (BayesFactor[α<β]).

**Table S13** Single nucleotide substitutions and their functional effects in the partial fragment of the exon 1 of *Mstnb* gene of catfish catfish populations in Thailand compare with the reference sequence (Accession number X NC_071104)

| **No** | **Allele-specific** | **Variable nucleotide position and nucleotide substitution** | **Mutation type** | **Amino acid change (substitution)** |
| --- | --- | --- | --- | --- |
| 1 | *CF_Mstnb*01* | 36,326,310 G → A | Missense | Val to Ile (GTT to ATT) |
| 2 | *CF_Mstnb*01–03, 07* | 36,326,319 A → G | Missense | Lys to Glu (AAG to GAG) |
| 3 | *CF_Mstnb*08* | 36,326,334 A → T | Nonsense | Arg to Stop (AGA to TGA) |
| 4 | *CF_Mstnb*01–03, 07* | 36,326,361 G → A | Missense | Ala to Thr (GCC to ACC) |
| 5 | *CF_Mstnb*03* | 36,326,367 A → G | Missense | Ser to Gly (AGT to GGT) |
| 6 | *CF_Mstnb*07* | 36,326,379 G → A | Missense | Val to Ile (GTT to ATT) |
| 7 | *CF_Mstnb*01–03, 07* | 36,326,391 C → T | Missense | His to Tyr (CAC to TAC) |
| 8 | *CF_Mstnb*01–03, 07* | 36,326,396 G → A | Missense | Arg to Lys (AGG to AAG) |
| 9 | *CF_Mstnb*01–08* | 36,326,406 T → C | Missense | Trp to Arg (TGG to CGG) |
| 10 | *CF_Mstnb*01–03, 07* | 36,326,426 A → C | Missense | Gln to His (CAA to CAC) |
| 11 | *CF_Mstnb*01–03, 07* | 36,326,469 T → C | Missense | Trp to Arg (TGG to CGG) |
| 12 | *CF_Mstnb*02* | 36,326,484 G → A | Missense | Val to Ile (GTC to ATC) |
| 13 | *CF_Mstnb*06* | 36,326,496 G → A | Missense | Val to Ile (GTC to ATC) |
| 14 | *CF_Mstnb*03* | 36,326,511 C → T | Missense | Arg to Cys (CGT to TGT) |
| 15 | *CF_Mstnb*03* | 36,326,517 G → T | Missense | Gly to Trp (GGG to TGG) |
| 16 | *CF_Mstnb*03* | 36,326,559 G → A | Missense | Val to Ile (GTC to ATC) |
| 17 | *CF_Mstnb*01, 02, 07* | 36,326,568 T → C | Missense | Stop to Arg (TGA to CGA) |
| 18 | *CF_Mstnb*01, 02, 07* | 36,326,579 T → G | Missense | Leu to Arg (CTG to CGG) |
| 19 | *CF_Mstnb*05* | 36,326,589 G → A | Missense | Gly to Arg (GGG to AGG) |
